# Integrative analysis of epigenetic and transcriptional interrelations identifies histotype-specific biomarkers in early-stage ovarian carcinoma

**DOI:** 10.1186/s13048-025-01676-5

**Published:** 2025-05-19

**Authors:** Hugo Swenson, Ella Ittner, Lucas Werner, Elisabeth Werner Rönnerman, Claudia Mateoiu, Anikó Kovács, Pernilla Dahm-Kähler, Ghassan M. Saed, Szilárd Nemes, Per Karlsson, Toshima Z. Parris, Khalil Helou

**Affiliations:** 1 Department of Oncology, Sahlgrenska Academy, Institute of Clinical Sciences, University of Gothenburg, Gothenburg, Sweden; 2 Department of Clinical Pathology, Region Västra Götaland, Sahlgrenska University Hospital, Gothenburg, Sweden; 3 Department of Obstetrics and Gynecology, Sahlgrenska Academy, Institute of Clinical Sciences, University of Gothenburg, Gothenburg, Sweden; 4 AstraZeneca, Gothenburg, Sweden; 5 Department of Obstetrics and Gynecology, Wayne State University School of Medicine, Detroit, MI USA

**Keywords:** Ovarian cancer, Gene expression, DNA methylation, Bioinformatics, Machine learning

## Abstract

**Background:**

Epithelial ovarian cancer (EOC) is a deadly and heterogenous disease comprising five major histotypes: clear cell carcinoma (CCC), endometrioid carcinoma (EC), low- and high-grade serous carcinoma (LGSC, HGSC), and mucinous carcinoma (MC). Despite this heterogeneity, EOC is often treated as a homogenous disease, and reliable screening tests are lacking. Although progress has been made, there is a pressing need for biomarkers to refine patient stratification, guide treatment, and improve outcomes. Here, we elucidated the relationship between DNA methylation and gene expression patterns in EOC to identify histotype-specific biomarkers.

**Methods:**

Differential DNA methylation and gene expression analyses were performed for 86 early-stage EOC samples after histopathological reclassification stratified by histotype. The correlation between DNA methylation and gene expression was examined, and histotype-specific biomarkers were identified. Hierarchical clustering and predictive machine learning modeling were employed to assess the performance of the histotype-specific biomarkers using four external cohorts.

**Results:**

EOC histotypes exhibited distinct epigenetic, transcriptional, and functional profiles, with candidate histotype-specific biomarkers such as *CTSE* and *VCAN* effectively distinguishing CCC, HGSC, and MC on the transcriptional level. Gene expression for the candidate biomarkers was found to be reproducible across external cohorts, with histotype-specific differences remaining homogenous.

**Conclusions:**

This study identified promising histotype-specific biomarkers for EOC using integrative transcriptomic and epigenomic analysis. Furthermore, these findings indicate that additional stratification or potential reclassification of the EC histotype is warranted in future studies.

**Supplementary Information:**

The online version contains supplementary material available at 10.1186/s13048-025-01676-5.

## Introduction

Ovarian cancer (OC) is an aggressive and heterogenous disease, ranking among the most lethal cancer types affecting women worldwide. Although OC only accounts for about 2% of female cancers, it is responsible for approximately 20% of cancer-related deaths in women [1, 2]. Survival rates for early-stage (stage I-II) exceed 90% with invasive surgery and platinum-based chemotherapy. However, early-stage disease is often asymptomatic and effective early detection methods are lacking. Therefore, many patients will be diagnosed at an advanced stage (stage III-IV), typically after menopause, where the expected survival is less than 30%. Moreover, patients that initially respond well to platinum-based chemotherapy often develop resistance after initial treatment, with approximately 20–25% and 70% of early- and late-stage patients experiencing recurrence, respectively [3].

As early detection and intervention of OC is directly linked to patient outcome, there is an urgent need to develop more effective diagnostic methods for early-stage diagnosis [4]. Various cancer types, including OC, have been associated with widespread epigenetic changes, i.e., global hypomethylation of the cancer genome and focal hypermethylation of the promoter region of tumor suppressor genes. Such changes take place even before carcinogenesis has occurred and are known to increase in frequency as the cancer progresses [5]. As DNA methylation is a chemically stable process present in the cell-free DNA of bodily fluids, DNA methylation-based biomarkers hold great potential for accessible tumor-based fingerprinting to detect early onset OC, without the need for invasive surgery [6].

Malignant epithelial ovarian cancers (EOC), representing 90% of OC cases, are further stratified into five histological subtypes: High-grade serous carcinoma (HGSC, 70%), low-grade serous carcinoma (LGSC, 5%), clear cell carcinoma (CCC, 10%), endometrioid carcinoma (EC, 10%), and mucinous carcinoma (MC, 3%). Each histotype showcases distinct methylation patterns, biological characteristics, incidence rates, morphology, and clinical outcome. Therefore, an accurate stratification of the EOC histotypes could guide treatment decisions and improve patient outcome [7]. Recent efforts in OC biomarker discovery have identified well-established markers such as the genes *BRCA1/2, HE4* and the protein CA125 [8, 9]. However, most studies have focused on the disease as a single, homogenous entity, thereby failing to account for heterogeneity between the histotypes on a biological level.

Effective OC classifiers require a sensitivity > 75% and specificity > 99.6% to achieve a positive predictive value of 10% (i.e., detecting one true OC case among 10 possible cases [10]). To overcome such stringent requirements, diagnostic models consisting of multiple individual genetic markers can be constructed to further enhance sensitivity and specificity. Several promising histotype-specific biomarkers have been proposed for EOC, including *WT-1* and p53 for HGSC, *MUC5AC* for MC or *ARID1A* and Napsin A for CCC [11–13]. Despite this, to date no comprehensive and reliable gene panels exist for EOC histotype stratification, highlighting the need for histotype-specific genetic markers to enhance classification at transcriptional or epigenetic levels. The aim of the study was to (1) evaluate the relationship between differential DNA methylation and gene expression, and (2) identify potential candidate histotype-specific biomarkers on either an epigenetic or transcriptional level. For this purpose, transcriptional and DNA methylation profiles for 86 early-stage EOCs were studied, followed by validation using external EOC cohorts.

## Methods

### Patient cohorts and data acquisition

To investigate epigenetic and transcriptional differences between EOC histotypes in early-stage (stage I-II) ovarian carcinoma, 96 cases from a prior study (GSE101109/Training cohort [14]) were reclassified by board-certified pathologists at Sahlgrenska University Hospital (Gothenburg, Sweden) using formalin-fixed, paraffin-embedded (FFPE) sections. The reclassification followed the 2020 World Health Organization (WHO) and International Federation of Gynecology and Obstetrics (FIGO) OC histological classification guidelines [15]. Samples with matching RNA sequencing (RNA-seq.fastq files), DNA methylation data (.idat files), and clinical data corresponding to the four main EOC histotypes, i.e., HGSC (*n* = 45), MC (*n* = 7), EC (*n* = 21), and CCC (*n* = 13; Tables 1 and 2) were included in the training cohort. Samples of the LGSC histotype were excluded from analysis due to their low prevalence (*n* = 2).

**Table 1:** Training and test cohort histotype and platform characteristics

Cohort name	Accession ID	CCC	EC	HGSC	MC	Year of publication	Platform
Training	GSE101109	13	21	45	7	2018	Illumina Hiseq2500 (SCR_016383)
Test	GSE2109	12	28	31	8	2005	Affymetrix Human Genome U133 Plus 2.0 Array
	GSE6008	8	37	39	13	2007	Affymetrix Human Genome U133 A 2.0 Array
	GSE44104	12	11	28	9	2014	Affymetrix Human Genome U133 Plus 2.0 Array
	E-MTAB-1814	17	19	16	15	2014	Agilent SurePrint G3 GE 8 × 60 k (A-GEOD-16083)

*CCC* Clear cell carcinoma, *EC* Endometrioid carcinoma, *HGSC* High-grade serous carcinoma, *MC* Mucinous carcinoma

The platforms used across cohorts highlight differences in data acquisition methods, which were accounted for in the analyses

**Table 2:** Clinicopathological features of the training cohort, stratified by histotype (*n* = 86)

Characteristic	All (*n* = 86)	HGSC (*n* = 45)	CCC (*n* = 13)	MC (*n* = 7)	EC (*n* = 21)
Age at diagnosis (years)					
Mean	63.8	65	62.3	61.7	62.6
Range	25–86	38–86	42–79	39–80	25–83
Stage					
I	56 (65.1)	25 (55.6)	11 (84.6)	6 (85.7)	14 (66.7)
II	30 (34.9)	20 (44.4)	2 (15.4)	1 (14.3)	7 (33.3)
CA125 (U/ml)					
U > 200	25 (29.1)	14 (31.1)	4 (30.8)	0	7 (33.3)
200 > U > 35	36 (41.9)	23 (51.1)	4 (30.8)	2 (28.6)	7 (33.3)
U < 35	25 (29.1)	8 (17.8)	5 (38.5)	5 (71.4)	7 (33.3)
Survival time (days)					
Mean	2732.9	2377.7	3135.5	2621.4	3282.0
Range	226–6473	239–5335	226–6473	366–5065	665–5576
Cause of death					
EOC	43 (50.0)	30 (66.7)	8 (61.5)	1 (14.3)	4 (19.1)
Other cancer	6 (7.0)	3 (6.7)	0	1 (14.3)	2 (9.5)
Other	26 (32.6)	9 (20.0)	5 (38.5.0)	4 (57.1)	8 (38.1)
Alive	11 (12.8)	3 (6.7)	0	1 (14.3)	7 (33.3)
Relapse					
Yes	31 (36.0)	17 (37.8)	4 (30.8)	4 (57.1)	6 (28.6)
Not Available	55 (64.0)	28 (62.2)	9 (69.2)	3 (42.9)	15 (71.4)
Adjuvant chemotherapy					
Platinum single	52 (60.5)	26 (57.8)	7 (53.9)	6 (85.7)	14 (66.7)
Platinum Combination	25 (29)	12 (26.7)	6 (46.2)	0	7 (33.3)
Non-platinum	8 (9.3)	7 (15.6)	0	1 (14.3)	0

Table shows n (% of total rounded up to one decimal point). *CCC* clear cell carcinoma; *EC* endometrioid carcinoma; *HGSC* high-grade serous carcinoma; *MC* mucinous carcinoma

Four external RNA expression datasets (Test cohorts: GSE2109, GSE6008, GSE44104, E-MTAB-1814 [16–18] of mixed EOC sample grade and stage, each containing at least 10 samples across 3/4 EOC histotypes in the training cohort were retrieved from the Gene Expression Omnibus (GEO, https://www.ncbi.nlm.nih.gov/geo/) or ArrayExpress (https://www.ebi.ac.uk/biostudies/arrayexpress) using the GEOquery (v.2.70 [19]) and ArrayExpress (v.2.9.0 [20]) packages in R/Bioconductor (v.4.3.0). Phenotypic annotations for test cohort datasets were retrieved using the MetaGxOvarian package (v.1.22.0 [21]). Datasets originating from the Affymetrix platform had their raw data files (.CEL) processed through the affy R package [22]. Processed data was normalized through the RMA algorithm (quantile normalisation) and control probes together with probes showing low intensity were removed before analysis. Datasets originating from the Agilent platform had their raw data processed through limma, where they were subjected to background correction followed by quantile normalization, removal of control probes together with low intensity probes, and the averaging of array replicate probes. Samples in dataset GSE2109 were reclassified according to the same FIGO guidelines used for the training cohort, whereas the other external datasets which use the 2014 FIGO classification had the “serous” histotype reclassified through deeming serous samples of grade 1 as LGSC, and serous samples of grade 2–3 as HGSC [23]. Any LGSC (serous grade 1) samples were removed from analysis. Finally, following PCA analysis, 6 samples were removed from dataset E-MTAB-1814 due to abnormal expression patterns (Fig. S1).

### RNA-seq quality control and read alignment

Computations using SNIC SENS resources were performed via the Uppsala Multidisciplinary Center for Advanced Computational Science (UPPMAX project ID sens2022542). Training cohort raw sequencing data files (.fastq) underwent adapter and quality trimming with the bbduk tool of the BBtools suite (BBtools v.38.08 [24]), quality assessment with FastQC (v.0.11.9 [25]) and summary compilation using MultiQC (v.1.12 [26]). Trimmed reads were aligned to the hg38 human reference genome (GRCh38.p13) with Ensembl genome annotation (Homo.sapiens.GRCh38.108) using the STAR RNA-seq aligner (v.2.7.9a [27]). Aligned samples were aggregated and assessed via MultiQC. Raw read counts were then obtained using the featureCounts tool of the subread package (subread v.2.0.0 [28]).

### Differential gene expression analysis

Differential gene expression (DGE) analysis was performed using R. For the training cohort, DESeq2 (v.1.40.1 [29]) was used to identify differentially expressed genes (DEGs) between the different histotypes. Lowly expressed genes were removed (*n* ≤7 samples with *n* ≤5 counts) and remaining data were subjected to variance stabilizing transformation. Genes were considered significant if they had a false discovery rate (FDR; Benjamini-Hochberg) adjusted *p*-value < 0.05 and absolute log_2_ Fold Change (FC) > 1.0 for histotype group comparisons. Genes without a corresponding Hugo Gene Nomenclature Committee (HGNC) symbol were excluded. Additionally, DEGs showing significant differential expression for a histotype compared to all other histotypes (e.g., for CCC: CCC-EC, CCC-HGSC, CCC-MC) were termed histotype-specific genes (HSGs). Performance was corroborated by jacknifing [30] for histotype group comparisons involving the smallest histotype group (MC) as reference.

### Functional annotation and oncogenic potential

Functional enrichment was performed for significant DEGs using goseq (v1.5.4 [31]) and biomaRt (v.2.58.2 [32]) for biological processes, and resulting gene ontology (GO) terms were subjected to multiple correction testing (overrepresented *p*-value; FDR). Significantly enriched GO-terms (adjusted *p*-value < 0.05) found in 2/3 possible DEG comparisons for a histotype were used as input for rrvgo (v.1.14.2 [33]) to identify parent GO terms. To evaluate the oncogenic potential of HSGs, the R package OncoScore (v.1.30.0 [34]) was applied to HSGs with HGNC symbols.

### Predictive modeling

Sample-level gene expression (log_2_(n+ 1) transformed counts) was used as predictors, with histotype classification as the response variable. Genes were chosen as predictor genes (PGs) for a histotype if they showed significant DGE in the training cohort for 2/3 comparisons when using the histotype of interest as reference. Optimal parameters for a XGBoost (XGB) model based on the training cohort were obtained through sequential grid search using the caret package (v.7.0–1 [35]) and feature selection was performed with the Boruta package (v.8.0.0 [36]). Selected features (PGs) for each histotype in the training cohort were combined into one feature-set, and for each dataset in the test cohort a fivefold cross-validation was conducted and repeated 100 times for a XGB classifier using test cohort data with optimal parameters and features for the training cohort. Binary (one vs. rest) classification models were constructed by defining the PG-associated histotype as one class (case), and all other histotypes as the second class (control), with feature selection, parameter optimization and cross validation performed through the same methodology as for the multiclass classification. Binary classification was then repeated for HSGs to evaluate differences in predictive performance.

### External DEG validation

The test cohort datasets were filtered to contain only samples associated with the 4 histotypes in the training cohort. Affymetrix microarray probes were annotated with affycoretools (v1.44.2 [37]) and Agilent microarray data with biomaRt. DGE analysis was performed for each dataset in the test cohort using limma (v.3.58.1 [38]) for each possible histotype comparison. Probes mapping to a gene were deemed significant for FDR < 0.05, absolute log_2_ FC > 0.585, and genes mapping to multiple probes were assigned to the probe with the lowest adjusted *p*-value.

### Differential methylation analysis

Training cohort DNA methylation microarray data was processed using the minfi package (v.1.48.0 [39]) and annotated by the Infinium MethylationEPIC v1.0 B5 Manifest File. Cross-reactive probes identified by the maxprobes package (v.0.0.2 [40]) as well as probes (1) with a detection *p*-value > 0.01, (2) a conversion rate < 80%, (3) overlapping SNP sites, (4) on the Y chromosome, and (5) with a beadcount < 5% were removed. Remaining probes were converted into beta-values (*β*) using minfi and filtered through the ChAMP package (v.2.32.0 [41]) champ.filter function (default parameters). Beta values for the remaining probes (*n* = 685,650) were normalized by Noob (minfi), followed by BMIQ (ChAMP). Differentially methylated probes (DMP) analysis was performed using ChAMP for each possible histotype group comparison. Probes were considered significant if they had Δβ > 0.2 and an adjusted *p*-value < 0.05. CpG sites were classified as hypermethylated when 0.7 < β-intensity, hemimethylated when 0.3 < β < 0.7, and hypomethylated when β < 0.3. Differentially methylated region (DMR) analysis was performed with the DMRcate (v.2.16.1 [42]) package pipeline for each possible histotype group comparison. Regions with adjusted *p*-value < 0.05, Δβ > 0.2 and n ≥ 5 overlapping CpG sites were deemed significant.

### Copy number analysis

Copy number aberrations (CNA) analysis was performed using methylation signal-intensity data with the conumee package (v.1.36.0 [43]). Sample-level segments and bins (50,000 bp region overlapping *n* > 15 CpG sites) were aggregated for each histotype group comparison. Bins exhibiting copy number aberrations across all histotype group comparisons for a specific histotype were compiled as histotype-specific bins. Conumee segment-results were then used as input for GISTIC2 (parameters: -conf_level 90, -genegistic, -ta 0.2 -td 0.2; v.2.0.23 [44]). Regions with FDR < 0.05 present in 25% or more of samples were deemed significant. Significant regions were compared to hg19 HSG coordinates (as the EPIC v1.0 array annotation maps to hg19) to assess associations between aberrant gene expression and focal/broad CNAs.

### Correlation between DNA methylation and RNA expression

DMPs and DMRs were remapped to hg38 coordinates using the Infinium MethylationEPIC v1.0 B5 Manifest file. Additionally, probes were categorized as mapping to opensea, gene-body or to the promoter region (2000 bp upstream, 50 bp downstream of transcriptional start site (TSS)). DNA methylation (β) was considered linearly correlated with gene expression if a probe overlapping a DEG was hypomethylated with a positive log_2_ FC (hypo-up), or hypermethylated with a negative log_2_ FC (hyper-down). DMR-DEG and DMP-DEG overlaps were categorized into four groups (hypo-up, hypo-down, hyper-up, hyper-down) according to the direction of *Δ*β for DMRs and log_2_ FC for DEGs. For CpG sites overlapping a DEG, sample-level CpG site β values were correlated to sample-level log_2_ counts. Genes with a Pearson correlation coefficient < −0.5 and adjusted *p*-value < 0.05 (Benjamini-Hochberg) were deemed significant.

## Results

### Patient and tumor characteristics

**Fig. 1:**
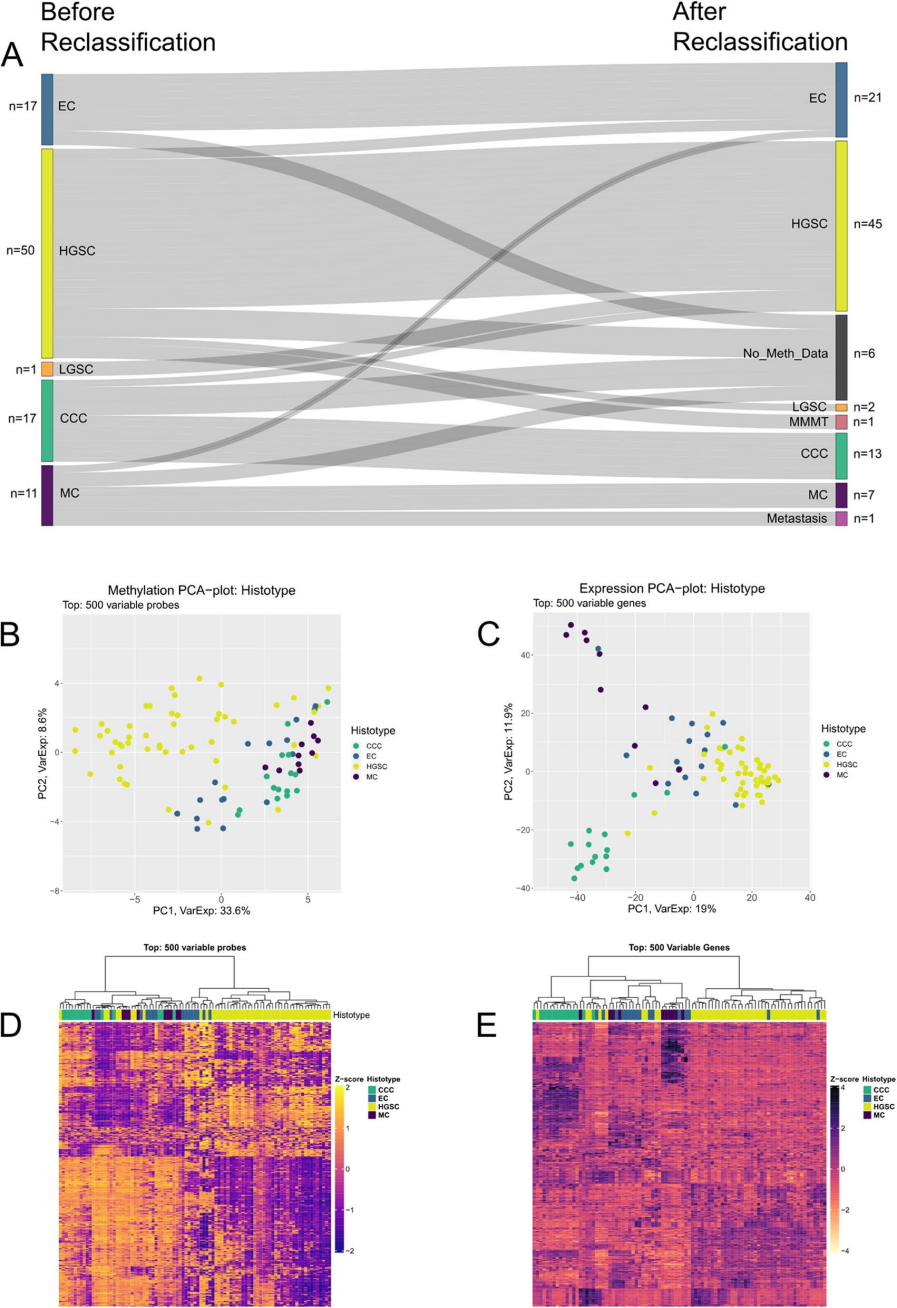
Reclassification of histotype for the training cohort and distinct epigenetic and transcriptional patterns in EOC by histotype. The figure illustrates the reclassification of the training cohort, and the epigenetic and transcriptional heterogeneity observed among EOC histotypes, except for EC which exhibits homogeneity. **A** Sankey diagram depicting the reclassification of EOC samples. **B** Principal component analysis (PCA*)* plot showing the top 500 most variable CPG site probes and (**C**) genes*.* Heatmap with hierarchical clustering (Euclidian distance, Ward.D2 clustering criterion) for the top 500 most (**D**) variable CpG probes and (**E**) genes*. CCC*: Clear cell carcinoma, *EC*: Endometrioid carcinoma, *LGSC*: Low-grade serous carcinoma, *HGSC*: High-grade serous carcinoma, *MC*: Mucinous carcinoma, *MMMT*:M alignant mixed Müllerian tumor, *Z-Score*: Statistical measure representing a value's relative position in relation to the mean of its group, *EOC:* Epithelial ovarian cancer

After histopathological reclassification, histotype changes were observed in 11/96 early-stage EOC samples (Fig. 1). In total, 90/96 reclassified EOC samples had matching DNA methylation data, of which 86 belonged to one of the four primary histotypes CCC, EC, HGSC, and MC. The mean age at diagnosis was 64 years (range; 25–86 years), with no substantial differences between the histotypes (Table 2). Only HGSC showed a near-equal distribution between stage I (*n* = 25) and stage II (*n* = 20), whereas the majority of CCC and MC samples were stage I (~ 85%). As expected, patients with stage I disease had higher 5-year survival rates (68%) than those with stage II disease (53%). With CCC exhibiting the highest 5-year survival rate (82%), followed by EC (71%) among stage I and II patients (Table S1).

### Differential gene expression analysis by histotype in the training cohort

After reclassifying the 86 EOC samples, we then conducted DGE analysis to identify histotype-specific molecular profiles. The highest number of DEGs was found between HGSC and CCC (*n* = 1314), and the lowest between EC and HGSC (*n* = 119; Table S2, Fig. S2). DEGs for HGSC were predominantly downregulated, whereas CCC exhibited predominantly upregulation compared to the other histotypes. In total, 655 PGs were found for CCC, 46 for EC, 224 for HGSC, and 325 for MC. CCC had the highest number of HSGs (*n* = 167), whereas EC had none (Table S3). HSGs for MC and CCC were predominantly upregulated, whereas those for HGSC showed mixed expression profiles (Fig. S3). Jackknifing revealed that DEG results were consistent for the removal of non-MC samples, and relatively consistent for MC samples except for one MC sample (Table S4).

### Functional annotation in the training cohort and oncogenic potential

GO analysis of DEGs revealed enriched biological processes (BP) present in multiple comparisons: DEGs for CCC were mainly involved in tissue development, metabolic processes (diterpenoid, retinoid, hormone), and multicellular processes. EC was primarily involved in metabolic processes (terpenoid, monocarboxylic, oxoacid and organic and hormone). HGSC was primarily involved in glucuronidation (cellular, flavonoid), metabolic processes (retinoid, nitrogen cycle), and hormonal regulation. MC was mainly involved in digestion, metabolic processes (lipid, oxoacid, alcohol, nitrogen cycle), microvillus regulation (length, organization) and lipid transport (Fig. S4).

Oncoscore analysis was applied to HSGs with HGNC symbols for CCC, HGSC, and MC. In total, 58/116 CCC, 17/19 HGSC, and 45/88 MC HSGs with a HGNC symbol passed the cut-off threshold (OncoScore ≥ 21.09; Table S5 - 7; Oncoscore: Relative measurement of articles mentioning a gene and cancer, to the total number of articles mentioning a gene) indicating an association with cancer based on existing literature. Additionally, 18 CCC, 5 HGSC and 12 MC HSGs had OncoScore > 50, among these were candidate cancer biomarkers such as *RNASET2* for CCC, *AKR1B10* for HGSC, and *KRT20* for MC.

Several gene families associated with cancer were found among HSGs. The solute carrier (*SLC)* gene family was identified as a key family of HSGs for CCC (*n* = 5), and to a lesser extent in MC (*n* = 2). Similarly, members of the mucin (*MUC*) gene family members (*n* = 3), known for their role known role as genetic markers in EOC, were identified as HSGs for MC. Members of the *UGT1A* family (*UGT1A1*, *UGT1A3*, *UGT1A6*, *UGT1A9*, and *UGT1A10*) were downregulated HSGs for HGSC, a trend that was consistent with DEG results in the test cohort.

### Predictive classification

Out of 40,570 genes in the training cohort with a corresponding gene symbol, external cohort gene coverage ranged between 32–57% of training cohort genes after preprocessing, filtering and annotation steps were carried out (Table S8). Multiclass predictive classification classified all histotypes with ~ 70% or higher sensitivity > 70% specificity and > 70% AUC in all test cohort datasets except for GSE2109, which performed worse for all histotypes. Multiclass feature panels varied in number of selected features due to coverage in the test cohort datasets, and consisted of 10–11 genes for CCC, 19-21 genes for EC. 21–24 genes for HGSC, and 11–15 genes for MC (Table S9). Out of the total 122 unique features found in all models, 30 were identified as HSGs in the training cohort. Binary models for all the test cohort datasets had mean sensitivity > 50%, mean specificity > 70%, and mean AUC > 70%. Binary models for HSGs showed similar predictive performance and number of features to binary models for PGs (Table S10 - 11).

### External DEG analysis and validation

**Fig. 2:**
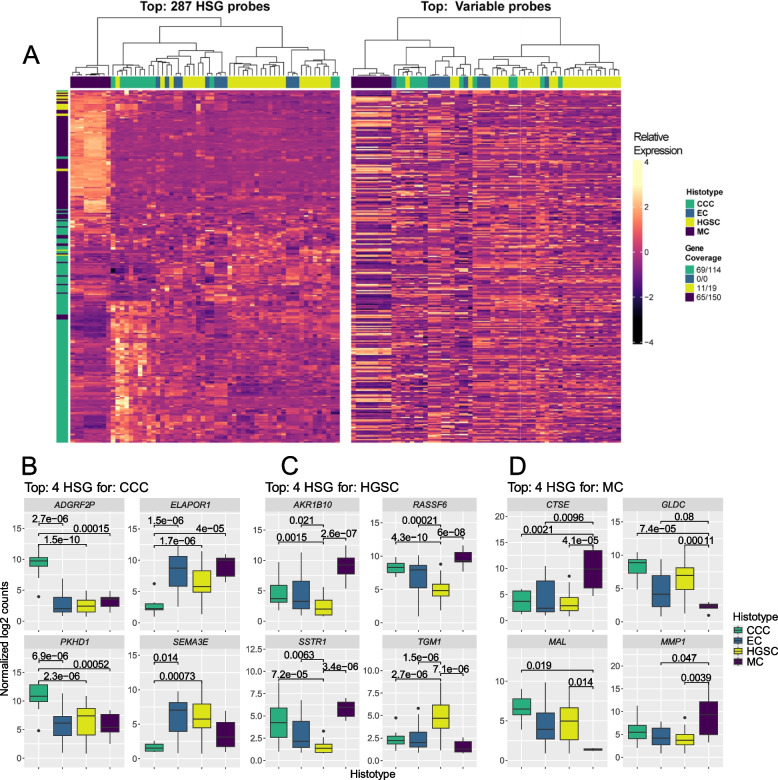
Hierarchical clustering of histotype-specific genes (HSGs) in the GSE44104 test cohort dataset and boxplots displaying expression for HSGs in the training cohort. **A** Heatmaps and hierarchical clustering (Euclidian distance, Ward.D2 clustering criterion) of expression data for 287 probes mapping to 145 HSGs: (left) HSGs with mapping probes in GSE44104, (right) the 287 most variable probes in GSE44104. Color mapping indicates z-score for gene expression, with genes and samples clustered separately. **B-D** Boxplots illustrating aberrant gene expression patterns (log_2_ normalized counts) in the training cohort for the top 4 HSGs for (**B**) CCC, (**C**) HGSC, and (**D**) MC showing between-group variance in expression. Values above boxplots represent Wilcoxon test *p*-values. *CCC*: Clear cell carcinoma, *EC*: Endometrioid carcinoma, *HGSC*: High-grade serous carcinoma, *MC*: Mucinous carcinoma, *Z-Score*: Relative measurement of a value in relation to the mean of a group of values to which it belongs, *Gene-coverage*: The number of HSGs with a matching probe in the dataset relative to the total number of HSGs

Hierarchical clustering paired with bootstrapping of expression data for HSGs revealed that MC and CCC more consistently formed separate clusters using HSGs compared to clustering by the most variable genes, whereas HGSC and EC samples showed similar expression profiles for HSGs for HGSC. Additionally, HSGs for MC and CCC were overexpressed relative to the other histotypes in datasets from the test cohort (Fig. 2, Fig. S5 - 13). HSGs were validated using external test cohort DEG results (Table S12). In total, 46/96 HSGs for MC, 39/167 HSGs for CCC, and 14/20 HSGs for HGSC were deemed DEGs in half or more test cohort datasets for comparisons with their associated histotype as reference, including 26/30 HSGs used in multiclass predictive models (Table S13 - 16).

### Differential methylation analysis

**Fig. 3:**
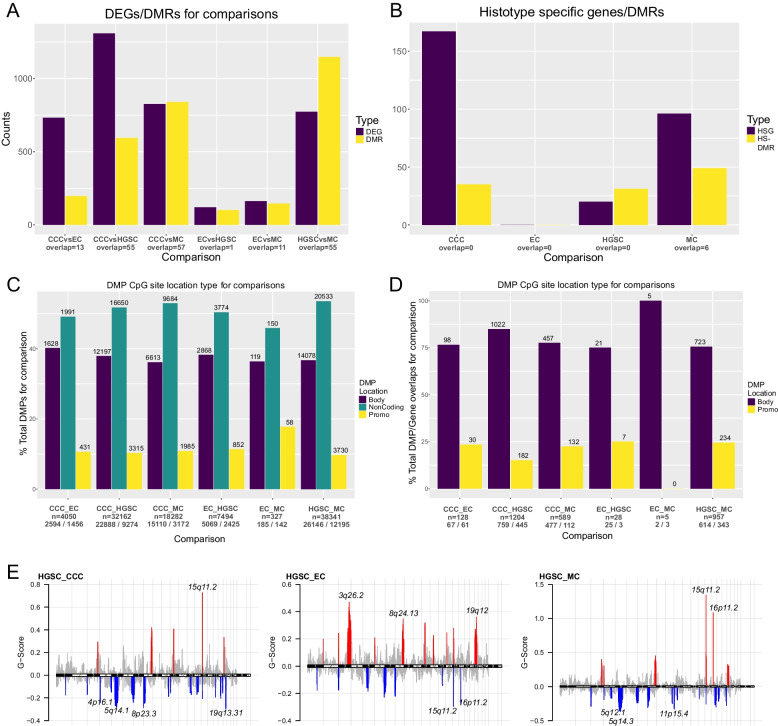
Overview of DMPs, DMRs, DEGs, and CNAs identified using the training cohort, highlighting the homogeneity of EC with respect to gene expression and DNA methylation, as well as the hypermethylation of CCC compared to the other histotypes. **A** DEGs/DMRs for each unique histotype contrast, **B** histotype-specific DEGs and histotype-specific DMRs, **C** DMP-site location type for unique histotype contrasts (promoter = 2000 bp upstream of TSS, 200 bp downstream of TSS), **D** DMP/DEG overlaps for each unique histotype contrast. Numbers above the bars represent the number of DMPs located in the gene body, non-coding regions, or promoter, while numbers below the contrast names indicate the total number of DMPs for each contrast (row 1) and DMP methylation type (Hypermethylated (δ *β* > *0.2*)/Hypomethylated (δ *β* < *− 0.2*); row 2). **E** GISTIC CNA analysis results for HGSC. Red peaks represent CNA gains, blue peaks represent CNA losses (labeled with cytoband name). *CCC*: Clear cell carcinoma, *EC*: Endometrioid carcinoma, *HGSC*: High-grade serous carcinoma, *MC*: Mucinous carcinoma, *G-score*: Score based on the aberration amplitude relative to its frequency across samples, as used by GISTIC*. DEG*: Differentially expressed gene, *DMP*: Differentially methylated probe, *DMR*: Differentially methylated region, *CNA*: Copy number aberration, *HSGs/HS-DMRs*: DEGs/DMRs found in all histotype comparisons, using the same histotype as reference

Differential methylation analysis of the training cohort histotypes showed that the HGSC-MC comparison yielded the highest number of DMPs (*n* = 38350) and DMRs (*n* = 772), while the MC-EC and HGSC-EC comparisons yielded the least number of DMPs (*n* = 327) and DMRs (*n* = 108), respectively (Table S17, Fig. S14 - 15). DMPs were primarily located in the gene-body and non-coding regions of the genome for all histotype comparisons, with probes overlapping the promoter region of a gene accounting for roughly 10% of DMPs. Similar to the results from the DEG analysis, MC and CCC were found to be the most heterogeneous with respect to the other histotypes. Moreover, CCC was shown to be comparatively hypermethylated, while MC was primarily hypomethylated (Fig. 3, Fig. S16 - 19). No chromosomal regions associated with DMPs or DMRs were overrepresented for the different histotypes. However, histotype-specific DMRs were found to be primarily located on chromosomes 4 (4/35 DMRs) and 12 (4/35) for CCC, chromosome 7 for HGSC (5/31) and chromosomes 1 (10/49), 10 (5/49), and 5 (5/49) for MC.

### Copy number analysis

Aggregation of sample-level CNA bins (50000 bp) revealed that HGSC had more bins showing gain or loss with a sample frequency > 30% compared to the other histotypes (Fig S20 - 23). Aberrant genomic regions (with respect to the other histotypes) identified by GISTIC 2.0 showed that CCC had no significant (frequency > 25%, FDR < 0.05) CNA regions compared to HGSC and EC, but had gains spanning chromosomes 20 and 17 when compared to MC. EC predominantly had genomic gains spanning chromosomes 1, 7, and 18 for all comparisons, while MC showed genomic losses spanning chromosomes 17, 8, and 6 for all histotype comparisons. HGSC showed a mixed profile with primarily losses across the genome. Additionally, CNAs spanning chromosomes 18, 17, 3, 20, and 13 were identified at a noticeably higher frequency for all histotype comparisons. For HGSC and CCC, DEGs overlapping significant CNA regions identified by GISTIC were predominantly copy number loss-downregulation (Table S18-19, Fig. S24-27). Amongst these genes were HSGs such as *VCAN* when HGSC was compared to CCC (11 HSGs for CCC, 3 for HGSC) and *KRT20* when HGSC was compared to MC (12 HSGs for MC, 1 for HGSC). For EC, overlapping DEGs displayed copy number loss-upregulation when compared to MC, while MC had no DEGs overlapping significant regions for matching histotype comparisons.

### Integrated transcriptomics and DNA methylation analysis

DMPs overlapping DEG genomic coordinates were found to primarily overlap with the gene body, a pattern also observed for HSGs. However, the number of DMP-gene overlaps varied across individual genes (ranging from 1 to 8 overlapping DMPs), with the highest number found for *CSGALNACT1* (*n* = 8). DEGs with multiple DMP sites displayed a mixed profile of methylation and expression (hypo-up, hyper-down, hypo-down, hyper-up). Generally, DMPs in the gene body and promoter region exhibited inverse methylation patterns to one another.

Although only a small fraction of DMPs in the histotype comparisons overlapped HSGs (*n* < 0.5%), many HSGs contained at least one DMP site within either the gene-body or promoter region (*n* = 0–45%). Moreover, the proportion of HSG-DMP site overlaps was consistently higher than the proportion of DEG-DMP overlaps across all histotype comparisons (Table S20). Few DEGs overlapped with DMRs in the same histotype group comparisons, with the highest number of overlaps found in the CCC and MC comparison (*n* = 57, 17.4% total DMRs, 6.9% total DEGs). The largest number of HSG-DMR overlaps occurred between HGSC and MC (*n* = 13, 1.7% total DMRs, 12.1% total HSGs for HGSC, MC). Overall, histotype comparisons involving MC exhibited the highest number of HSGs overlapping DMRs (Table S21).

**Fig. 4:**
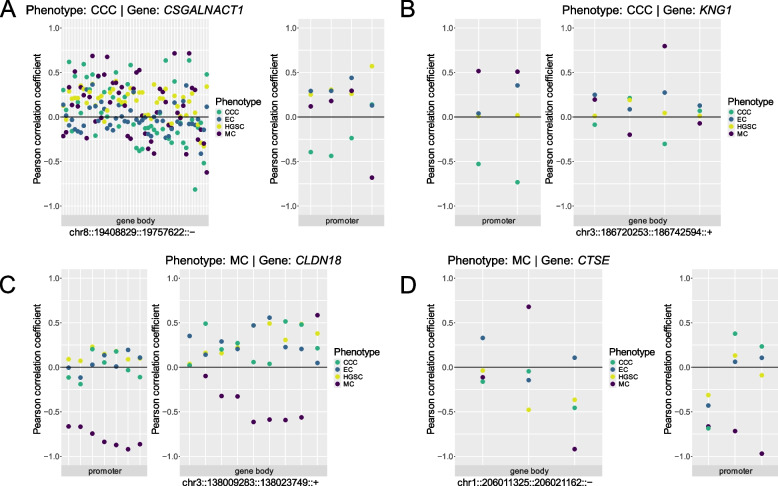
Correlation between DNA CpG site methylation and gene expression for validated HSGs. Dots represent the Pearson correlation coefficient between mean methylation (*β)* for CpG sites overlapping HSG coordinates and gene expression (log_2_ normalized counts). A negative correlation coefficient indicates a linear association between methylation and expression (hypermethylation-downregulation, hypomethylation-upregulation). HSGs for (**A**,** B**) CCC and (**C**,** D**) MC. *Promoter*: Promoter region (2000 bp upstream of the transcriptional start site (TSS), 200 bp downstream of TSS). *CCC*: Clear cell carcinoma, *EC*: Endometrioid carcinoma, *HGSC*: High-grade serous carcinoma, *MC*: Mucinous carcinoma

Correlation analysis of DNA methylation (β-values) for CpG sites overlapping HSG genomic coordinates with sample-level normalized log2(n + 1)-scaled counts showed that CCC had the highest number of HSGs (*n* = 20) with significantly correlated CpG sites in the promoter region (e.g., *CLDN18* and *CDHR5*). HSGs with n ≥ 1 significantly correlated CpG sites in the promoter region predominantly showed a negative correlation between gene expression and DNA methylation across the entire promoter region, in contrast, the gene body exhibited both positive and negative correlation (Fig. 4, Fig. S28 - 29; Table S22).

## Discussion

To investigate differences between EOC histotypes on the epigenetic and transcriptional levels, we performed a comprehensive analysis of RNA expression and DNA methylation data for 86 early-stage EOCs reclassified using FFPE sections and stratified by histotype. For the training cohort, a comparison between the DEG and DMP/DMR results before and after histotype reclassification (using the same pipeline and parameters) revealed an increase in the number of DEGs in all histotype comparisons (18–62% more DEGs after reclassification). Significant DMPs and DMRs also increased after reclassification except for the HGSC-EC comparison, suggesting reclassification led to improved separation of the histotype groups at both an epigenetic and transcriptional level in the training cohort. Integrated DGE and differential methylation analyses in the training cohort demonstrated that most EOC histotypes exhibited distinct heterogeneity on both an epigenetic and transcriptional level. Genes differentially expressed for one histotype compared to others in the training cohort were found to have a higher number of epigenetic aberrations in the promoter region compared to other DEGs. And successfully stratified histotypes through both hierarchical clustering and predictive classification in external cohorts. With subsequent DGE analysis revealing reproducibility in expression patterns across external cohorts.

For the training cohort, EC displayed homogeneity with MC for DNA methylation and with HGSC and MC for RNA expression. MC was characterized by hypomethylation and overexpression compared to the other histotypes in the training cohort, except for CCC, and showed similar expression patterns in the test cohort datasets. DEG results for comparisons involving EC showed the most variation across the training and test cohort datasets. This pattern was also seen in the PCA plots for the training and test cohorts, where EC had the highest degree of within-group heterogeneity in both the training and test cohorts, and EC samples were often found in clusters containing other histotypes. HGSC was found to have the highest mortality attributed to OC (66.7%), potentially reflecting its more aggressive clinical behavior compared to the other histotypes. DEGs for HGSC were found to be downregulated in both the training and test cohort datasets, and HSGs for HGSC included genes either directly or indirectly associated with tumor suppression such as *RASSF6* and *AKR1B10* through its interaction with p53 [45, 46]. This raises the question of whether the aggressive clinical behavior for HGSC is in part due to the relative downregulation of genes involved in tumor suppression.

Oncogenic assessment using Oncoscore confirmed that several HSGs identified in our analyses have an established association with cancer in the existing literature. While most of these genes have been linked to OC or cancer more broadly, specific associations with EOC histotypes were rare [47, 48]. Further literature review of HSGs revealed that many belong to gene families previously associated with carcinogenesis and/or EOC, including *ARID, CLDN, MUC, KLK*, and *SLC*. CCC exhibited a relatively high number of HSGs from the *SLC* gene family, while MC had a higher prevalence of HSGs from the *MUC* gene family. The downregulation of several *UGT1A* family members in HGSC is particularly interesting due to their established link to carcinogenesis and therapy-induced toxicity [49, 50].

GO enrichment analysis showed that 3/4 histotypes had DEGs involved in the metabolic processes of terpenoids for > 2/3 possible histotype comparisons. In particular, HGSC was characterized by enriched BP in cellular and flavonoid glucuronidation, likely due to the high prevalence of differentially expressed *UGT*-genes which are directly involved in the glycosylation of secondary metabolites. As terpenoids and flavonoids are known to have anti-cancer properties and *UGTs* are known to influence drug resistance and cancer progression [51, 52]. The aberrant expression of genes involved in glucuronidation, and the metabolism of exogenous compounds could potentially influence EOC carcinogenesis and patient response to therapy. CCC was characterized by BP in tissue development, and MC was found to have many enriched BP categories involved in lipid digestion and metabolic processes, suggesting potential pathways driving histotype-specific tumor behavior.

Predictive classifiers created from training set PGs were able to stratify EOC histotypes through both binary and multiclass classification. Binary models showed similar performance for PGs and HSGs, and while only a select few HSG models achieved the suggested thresholds for OC classifiers (sensitivity > 75%, specificity > 99.6%; GSE6008 for CCC & MC, GSE44104 for MC), all binary HSG models showcased AUC > 0.8 except for CCC for GSE44104. The high representation of HSGs in multiclass models and overall predictive performance for both binary and multiclass classification tasks indicate that HSGs used in these models still hold potential for histotype stratification in EOC. Inspection of PCA plots for the test cohort datasets showed that EC samples clustered together with HGSC samples for HSGs, and that in datasets where predictive performance was lower for a histotype, that same histotype exhibited poor within-group homogeneity. Suggesting predictive performance was partially hindered by a lack of distinguishable expression profiles for those samples. It remains unclear whether this is due to biological variability such as cell type purity or pathological grade, or inconsistencies in histotype classification as the test cohort datasets varied considerably in their year of origin. Notably, histotype classifications of dataset GSE2109 were reclassified by the first author in accordance with the 2020 WHO and FIGO guidelines. This resulted in an increase in sensitivity for predictive modeling results in the binary classification tasks for CCC (from 0.29 to 0.61) and MC (from 0.26 to 0.74) compared to the histotype classifications available on GEO.

Results for the DEG analysis performed for both the training cohort and for the test cohorts may have seen the statistical outcome of the results influenced by the low number of samples for some groups, and the imbalance of the number of samples associated with a histotype. Similarly, classification models may have experienced overfitting due to the limited number of predictors resulting from poor gene coverage, or overfitting due to the small number of response variables (samples). Another important consideration is that the test cohort datasets originate from different research groups, which implies variability in protocols for extraction, quality control, quantification, geographical locations, seasonal factors (e.g., humidity and temperature during extraction) which could all influence the data. Furthermore, as bulk RNA-seq offers higher sensitivity and specificity when compared to the microarray technologies used by the test-cohort datasets [53], and many HSGs lacked a corresponding probe in one or more microarrays. Technical differences in the platform used to generate data and their corresponding gene annotation likely impacted external DEG analysis and predictive classification negatively due to differences in resolution and missingness.

Finally, while efforts were made to homogenize the datasets through employing a similar bioinformatical pipeline for pre-processing the data and through post-hoc reclassification of EOC serous samples, the histopathological assessment of samples in test cohort datasets remain unknown to us and could influence the statistical outcome of the analysis due to differences in the classification guidelines used. Taken together, the influence of such confounding factors on the results cannot be easily dismissed [54]. Larger datasets with higher resolution, greater gene annotation coverage, consistent protocols for data generation and an even distribution of EOC histotype samples could in theory improve the statistical validity of the findings presented in the study, but to our knowledge, such a cohort does not exist in publicly available repositories.

To evaluate whether HSG expression patterns were consistent in other cohorts, hierarchal clustering and external DEG analysis was conducted. Hierarchical clustering revealed that HSGs could more effectively distinguish between histotype groups compared to clustering based on the most variable genes in both the training and test cohorts. Clusters generated using HSGs demonstrated better separation of histotypes and higher reproducibility in both the training cohort and the test cohort datasets. This effect was most pronounced for CCC and MC, as the number of HSGs for HGSC was comparatively low.

Most HSGs in the training cohort were classified as significant DEGs for the same histotype comparisons in the test cohort datasets. Additionally, the directionality of the aberrant gene expression in the training dataset was found to be homogenous with the test cohort datasets for the associated histotype comparisons. Out of the 30 HSGs found in the multiclass models (based on feature selection in the training cohort) 26 were found as DEGs in half or more of the external cohorts. With several HSGs such as *ARID3A*, *RNASET2* and *VCAN* for CCC, *RASSF6* and *UGT1A6* for HGSC and KRT20, *CDHR2,5* and *AGMAT* for MC found as DEGs in more than half of all external DEG comparisons for its associated histotypes. Indicating a high degree of reproducibility for HSG expression across multiple datasets despite sources of variability, and potential as biomarkers for histotype stratification in EOC. HSGs that were not identified as DEGs in the test cohort datasets were often found to have the characteristics of a DEG but failed the adjusted *p*-value cutoff, or to lack mapping probes in several of the external cohort datasets. As paired healthy ovarian tissue samples were not available in the training or test cohort, further comparisons of HSG expression in healthy and cancer tissue should ideally be done to evaluate the potential of HSGs as potential biomarkers for both histotype stratification and EOC detection.

Investigation of the epigenetic landscape of EOC in relation to the transcriptional profiles showed that while few DMPs overlapped DEG genomic coordinates, a relatively high proportion of DEGs corresponded to DMPs. A majority of overlapping DMPs were in promoter regions, especially for HSGs where they displayed a negative correlation between DNA methylation and gene expression. Whereas the gene body of HSGs instead showed a mix of negatively and positively correlated CpG sites, the latter adhering to the observed relationship between methylation of the gene-body and upregulated gene-expression [55]. As intronic regions experience a lower intensity of DNA-methylation when compared to exonic regions, even when having comparable GC content, and the probes mapping to CpG sites in the Infinium EPIC array are not evenly distributed [56, 57]. The observed variability in correlation between gene expression and DNA methylation of the gene body when compared to the promoter region may be due to the methylation of intragenic CpG rich intronic and exonic regions. The frequency of DEGs and to an even higher extent HSGs overlapping DMPs suggest that promoter region methylation aberrations may have a direct role in the dysregulation of gene expression in several HSGs.

Several DEGs for HGSC (and to a lesser extent for CCC) were located in regions with copy number losses, with a vast majority of these genes being downregulated. These observations underscore the influence of CNAs on DGE in HGSC, consistent with existing literature [58]. The relatively low sample size in the training cohort for all histotypes except HGSC likely limited the number of statistically significant regions identified by GISTIC (leading to the low number of DEG-CNA regions for EC and MC). DMRs did not overlap with DEGs as frequently as DMPs, likely due to the more stringent criteria for significance for DMRs. As many genes contained relatively few (*n* < 5) overlapping CpG sites, the number of possible DMR/DEG overlaps was reduced.

## Conclusion

Taken together, the present study demonstrates that HSG gene expression can be used to stratify EOC histotypes using both binary and multiclass predictive classification, as well as hierarchical clustering. We further show that HSGs are highly represented for cancer in existing literature, and that their expression patterns are reproducible across multiple cohorts, highlighting their potential as genetic markers for EOC histotypes. Although no large-scale histotype-specific relationship between epigenetic regulation and gene expression was observed for the training cohort, the negative correlation between promoter region methylation and gene expression identified in several HSGs suggests that epigenetic regulation may play a role in aberrant gene expression. Additionally, some HSGs (e.g., *CLDN18* and *KNG1*) demonstrated histotype specificity patterns of promoter methylation, indicating their potential as histotype-specific epigenetic biomarkers. These biomarkers could be detected on an epigenetic level without requiring invasive surgical procedures, warranting further investigation. Functional analysis and literature search suggest that genes involved in glucuronidation, and metabolism of exogenous compounds are associated with HGSC patient outcome, with the *UGT* gene family being of special interest as they were downregulated compared to all other histotypes in both training and test cohorts. Finally, the high degree of similarity between the EC and HGSC histotype in the training cohort and the heterogeneity in DEG results for EC observed in the test-cohort raises the question of whether further classification of EC is needed to improve the accuracy of subsequent bioinformatics analysis.

## Supplementary Information

Supplementary Material 1.

## Data Availability

All sequence data used in the study are available under their corresponding GEO or ArrayExpress accession codes. GEO: GSE101109, GSE2109, GSE6008, GSE44104 ArrayExpress: E-MTAB-1814
