# Supplementary material for: Integrative analysis of epigenetic and transcriptional interrelations identifies histotype-specific biomarkers in early-stage ovarian carcinoma
Source: J Ovarian Res. 2025 May 19;18:103. doi: 10.1186/s13048-025-01676-5 (PMC12087105; doi:10.1186/s13048-025-01676-5)
Supplement: Supplementary file 1 — Supplementary Material 1. [file 13048_2025_1676_MOESM1_ESM.pdf]

Supplementary Information (SI)

Article title

Integrative analysis of epigenetic and transcriptional interrelations identifies histotype-specific biomarkers in early-stage ovarian carcinoma

Journal

Journal of Ovarian Research

Authors

Hugo Swenson, Ella Ittner, Lucas Werner, Elisabeth Werner Rönnerman, Claudia Mateoiu, Anikó Kovács, Pernilla Dahm-Kähler, Ghassan M. Saed, Szilárd Nemes, Per Karlsson, Toshima Z. Parris, and Khalil Helou

Corresponding author

Hugo Swenson, hugo.swenson@gu.se

Figure 1 PCA plot of E-MTAB-1814 before and after removal of samples showing aberrant expression patterns

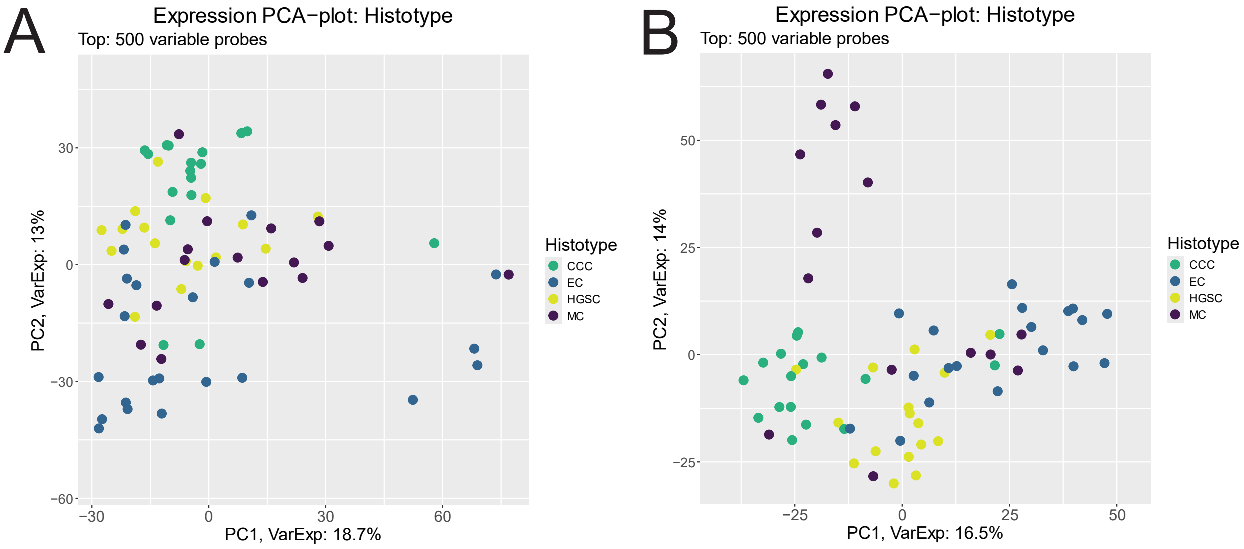

A: PCA plot of E-MTAB-1814 before removal of samples showing aberrant expression patterns (right side of plot)
B: PCA plot of E-MTAB-1814 after removal of samples showing aberrant expression patterns *CCC*: Clear cell carcinoma, *EC*: Endometroid carcinoma, *HGSC*: High-grade serous carcinoma, *MC*: Mucinous carcinoma

Table 1 5-year survival rates with respect to stage and histotype

Survival (years)	CCC	EC	HGSC	MC
**Stage I**	n=11	n=14	n=25	n=6
1	1	1	0.96	1
2	0.91	1	0.96	0.67
3	0.91	0.93	0.84	0.67
4	0.91	0.93	0.68	0.67
5	0.82	0.64	0.64	0.67
**Stage II**	n=2	n=7	n=20	n=1
1	0.5	1	1	1
2	0.5	0.86	0.95	1
3	0.5	0.86	0.85	0
4	0.5	0.86	0.6	0
5	0.5	0.71	0.5	0

CCC: Clear cell carcinoma, *EC*: Endometroid carcinoma, *HGSC*: High-grade serous carcinoma, *MC*: Mucinous carcinoma

Table 2 The number of differentially expressed genes between the OC histotypes in the training cohort

Comparison group	Total	Upregulated	Downregulated
EC – CCC	733	328	405
HGSC – CCC	1314	616	698
MC – CCC	797	390	407
CCC – EC	731	406	325
HGSC – EC	130	32	98
MC – EC	148	119	29
CCC – HGSC	1309	698	611
EC – HGSC	119	100	19
MC – HGSC	745	445	300
CCC – MC	826	442	384
EC – MC	162	43	119
HGSC – MC	772	327	445

Upregulated: (log2FC > 1.0, Benjamini-Hochberg< 0.05), *Downregulated*: (log2FC < -1.0, Benjamini-Hochberg < 0.05), *CCC*: Clear cell carcinoma, *EC*: Endometroid carcinoma, *HGSC*: High-grade serous carcinoma, *MC*: Mucinous carcinoma, *HSG*: Histotype specific gene (classified as significantly differentially expressed gene in 3/3 possible histotype comparisons for a histotype of interest used as reference in comparisons)

Figure 2 Venn diagram of DEGs in histotype comparisons in the training cohort

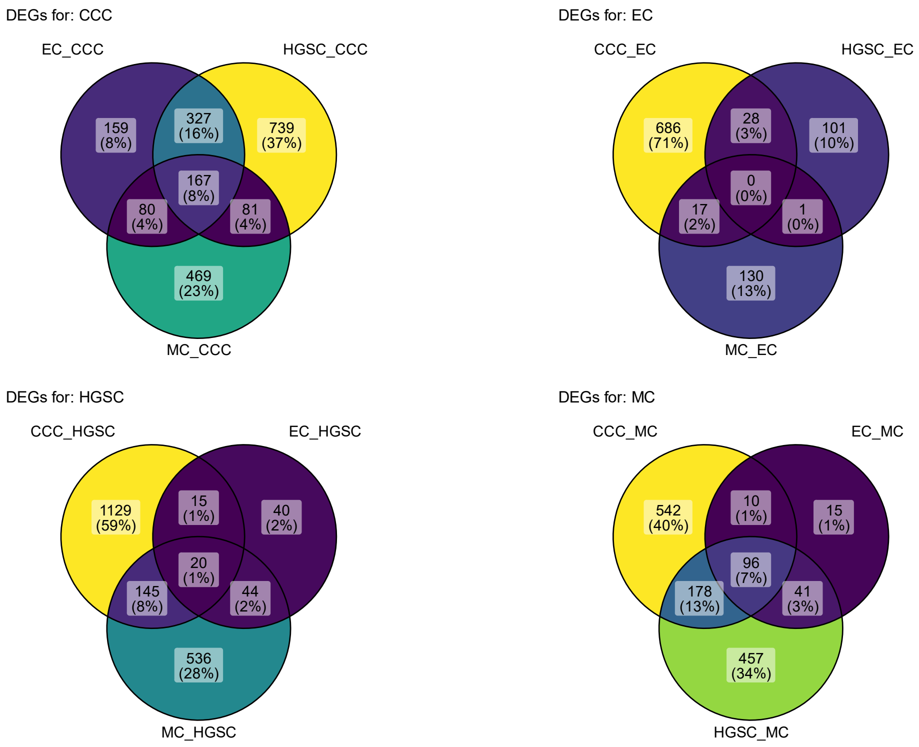

Venn-diagram of DEG-results for histotype comparisons in training cohort. *DEG*: Differentially expressed gene, *CCC*: Clear Cell Carcinoma, *EC*: Endometrioid Carcinoma, *HGSC*: High Grade Serous Carcinoma, *MC*: Mucinous Carcinoma. Plots created in r-package "ggVennDiagram"

Table 3 The number of differentially expressed HSGs for each histotype in the training cohort

**Histotype**	**Total**	**Upregulated**	**Downregulated**
CCC	167	144	23
EC	0	0	0
HGSC	20	4	16
MC	96	86	10

Upregulated: (log2FC > 1.0, Benjamini-Hochberg< 0.05), *Downregulated*: (log2FC < -1.0, Benjamini-Hochberg < 0.05), *CCC*: Clear cell carcinoma, *EC*: Endometroid carcinoma, *HGSC*: High-grade serous carcinoma, *MC*: Mucinous carcinoma, *HSG*: Histotype specific gene (classified as significantly differentially expressed gene in 3/3 possible histotype comparisons for a histotype of interest used as reference in comparisons)

Figure 3 Most variable HSGs in the training cohort

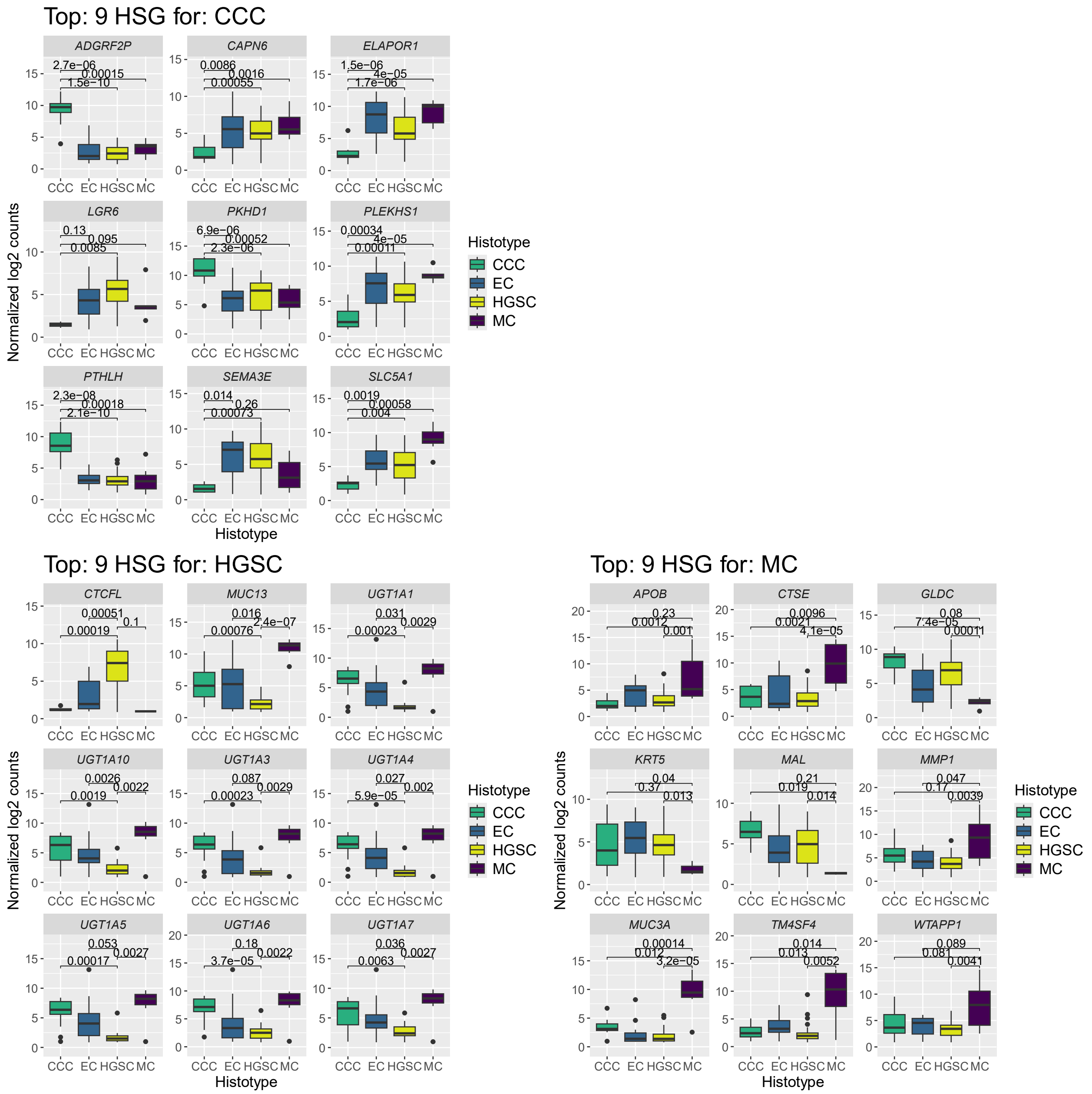

Most variable HSGs in training cohort for each histotype. Values over boxplots indicate wilcoxon test p-value. *CCC*: Clear cell carcinoma, *EC*: Endometrioid carcinoma, *HGSC*: High grade serous carcinoma, *MC*: Mucinous carcinoma, *HSG*: Histotype specific gene (classified as significantly differentially expressed gene in 3/3 possible histotype comparisons for a histotype of interest used as reference in comparisons). Plots created in r-package "ggplot"

Table 4 Jackknifing results for MC samples on histotype comparisons using MC as reference. Jackknifing for non-MC samples not shown individually

Sample	CCC vs. MC	EC vs. MC	HGSC vs. MC
MC 1	437	59	318
MC 2	402	38	269
MC 3	607	256	494
MC 4	392	32	256
MC 5	363	29	205
MC 6	379	31	212
MC 7	389	41	273
DEG Range MC samples	363-607	29-256	205-494
DEG Range no MC samples	419-477	44-81	325-358

DEG range: Lowest to highest number of DEGs found when performing jackknifing for DEG analysis. *CCC*: Clear cell carcinoma, *EC*: Endometroid carcinoma, *HGSC*: High-grade serous carcinoma, *MC*: Mucinous carcinoma

Figure 4 GO-term treemap of parent terms for significantly enriched DEG GO (BP) categories

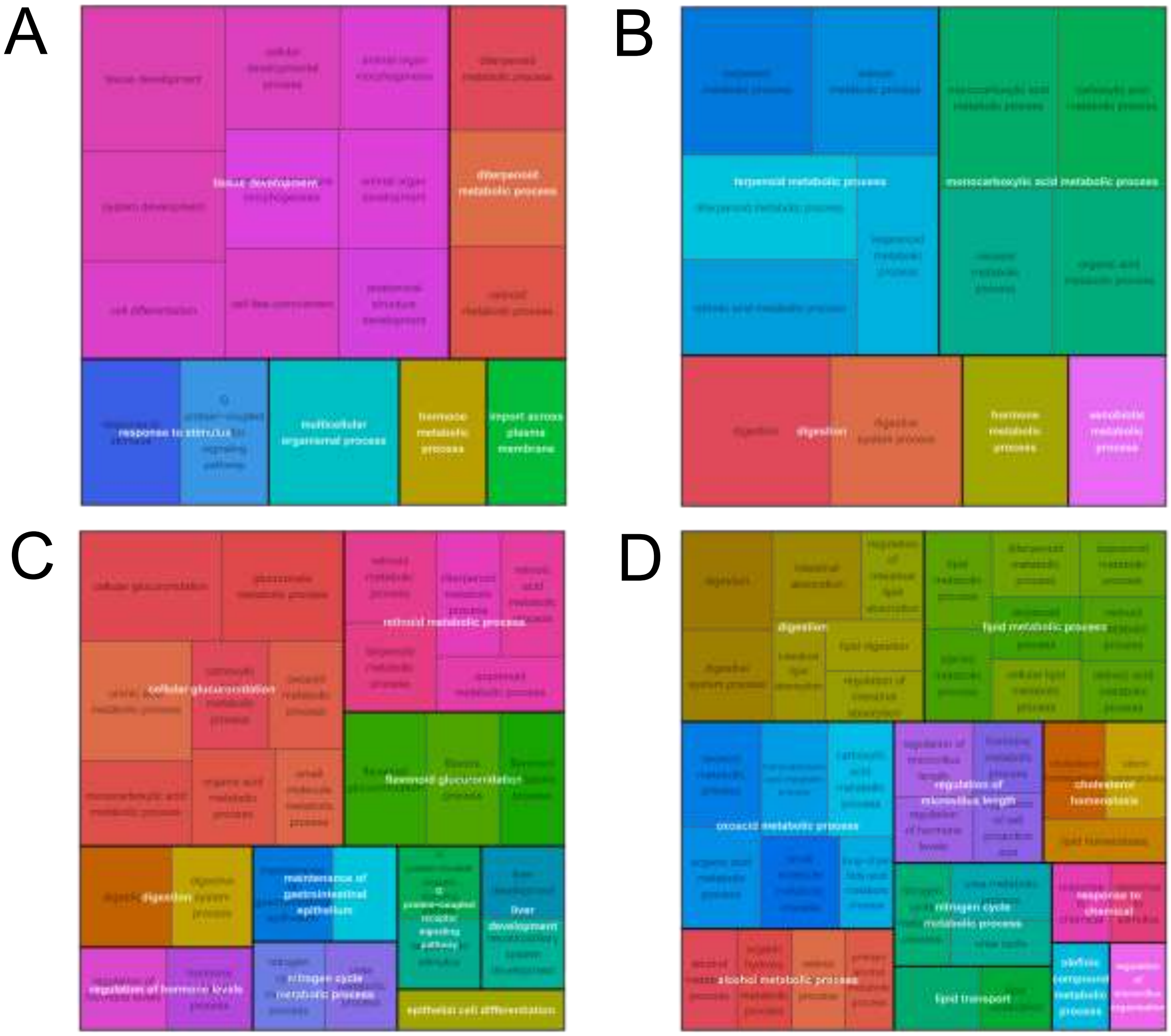

GO-term (BP) treemap of parent terms for DEG GO (BP) categories significantly enriched (adjusted overrepresented pvalue < 0.05) in 2/3 comparisons using the same histotype as reference in the training cohort, **(A):** Parental GO BP terms for DEGs in 2/3 CCC comparisons, (**B**)**:** Parental GO BP terms for DEGs in 2/3 *E*C comparisons, (**C**)**:** Parental GO BP terms for DEGs in 2/3 HGSC comparisons, **(D):** Parental GO BP terms for DEGs in 2/3 MC comparisons. *CCC*: Clear cell carcinoma, *EC*: Endometrioid carcinoma, *HGSC*: High grade serous carcinoma, *MC*: Mucinous carcinoma. Plots created in r-package "rrvgo"

Table 5 OnscoScore results for CCC HSGs

Gene	Gene citations	Cancer-associated citations (%)	Oncoscore
COLCA1	20	100	76.86
DRAIC	38	94.74	76.69
TTN-AS1	67	88.06	73.54
NAPSA	189	83.07	72.08
PLEKHS1	23	91.30	71.12
ELAPOR1	22	90.91	70.52
POU2AF3	7	100	64.38
PRIMA1	168	73.21	63.31
FBXO8	10	90	62.91
PTHLH	1454	68.64	62.11
VCAN-AS1	9	88.89	60.85
VWA2	28	75	59.40
RAB42	18	77.78	59.13
LINC01671	5	100	56.93
CPA4	64	67.19	55.99
PGBD5	29	68.97	54.77
BATF3	378	60.32	53.27
CPNE8	27	66.67	52.65
LINC00598	8	75	50

HSGs with OnscoScore > 50 (Cutoff for significance: Oncoscore > 21.09). *HSG*: Histotype specific gene, *CCC*: Clear cell carcinoma, *Oncoscore*: Relative measurement of articles mentioning a gene and cancer, to the total number of articles mentioning a gene, *HSG*: Histotype specific gene (classified as significantly differentially expressed gene in 3/3 possible histotype comparisons for a histotype of interest used as reference in comparisons)

Table 6. OnscoScore results for HGSC HSGs

Gene	Gene citations	Cancer-associated citations (%)	Oncoscore
RASSF6	65	92.31	76.98
CTCFL	170	83.53	72.26
AKR1B10	432	77.08	68.28
CDH17	245	74.69	65.28
MUC13	184	62.5	54.19

HSGs with OnscoScore > 50 (Cutoff for significance: Oncoscore > 21.09). *HSG*: Histotype specific gene, *HGSC*: High grade serous carcinoma, *Oncoscore*: Relative measurement of articles mentioning a gene and cancer, to the total number of articles mentioning a gene, *HSG*: Histotype specific gene (classified as significantly differentially expressed gene in 3/3 possible histotype comparisons for a histotype of interest used as reference in comparisons)

Table 7. OnscoScore results for MC HSGs

Gene	Gene citations	Cancer-associated citations (%)	Oncoscore
HHLA2	152	96.05	82.80
KRT20	767	90.87	81.39
GSDMC	96	88.54	75.10
CLDN18	366	84.97	74.99
ANXA10	120	85	72.69
VSIG1	21	90.48	69.88
PRIMA1	168	73.21	63.31
TM4SF4	32	78.13	62.5
RAPGEFL1	6	100	61.32
WTAPP1	9	88.89	60.85
GALNT5	19	73.68	56.34
MUC12	86	61.63	52.04

HSGs with OnscoScore > 50 (Cutoff for significance: Oncoscore > 21.09). *HSG*: Histotype specific gene (classified as significantly differentially expressed gene in 3/3 possible histotype comparisons for a histotype of interest used as reference in comparisons), *MC*: Mucinous carcinoma, *Oncoscore*: Relative measurement of articles mentioning a gene and cancer, to the total number of articles mentioning a gene

Table 8 Gene and HSG coverage in test-cohort datasets

Name	Gene coverage (%)	HSG coverage (%)
GSE6008	12887/40570 (32%)	117/282 (41%)
GSE44104	19100/40570 (47%)	162/282 (57%)
GSE2109	19890/40570 (49%)	162/282 (57%)
E-MTAB-1814	22953/40570 (57%)	187/282 (66%)

Gene Coverage: Total number of probes mapping to a gene with a gene symbol, in relation to the total number of genes with gene symbol in the training cohort *HSG*: Histotype specific gene (classified as significantly differentially expressed gene in 3/3 possible histotype comparisons for a histotype of interest used as reference in comparisons)

Table 9 Predictive classification statistics for multiclass models using PGs

Cohort	Histotype	Sensitivity	Specificity	Model AUC	Features
GSE6008	CCC	0.94	0.99	0.92	11
GSE6008	EC	0.74	0.81	-	20
GSE6008	HGSC	0.76	0.89	-	24
GSE6008	MC	0.89	0.96	-	12
GSE44104	CCC	0.76	0.95	0.96	10
GSE44104	EC	0.82	0.92	-	21
GSE44104	HGSC	0.89	0.92	-	23
GSE44104	MC	0.97	1	-	15
E-MTAB-1814	CCC	0.76	0.91	0.85	11
E-MTAB-1814	EC	0.71	0.91	-	19
E-MTAB-1814	HGSC	0.63	0.9	-	21
E-MTAB-1814	MC	0.75	0.87	-	12
GSE2109	CCC	0.54	0.9	0.79	11
GSE2109	EC	0.58	0.76	-	20
GSE2109	HGSC	0.68	0.84	-	21
GSE2109	MC	0.63	0.96	-	11

Mean values for cross-validated XGB classification (folds=5, resamples=100) constructed through PGs. *Sensitivity*: True positives/(True positives + False negatives), *Specificity*: True negatives/(True negatives + False positives)), *AUC*: Area under ROC curve, *Features*: Genes used as predictors in classifier model, *PG*: Predictor Gene (classified as significantly differentially expressed gene in 2/3 possible histotype comparisons for a histotype of interest used as

reference in comparisons). *CCC*: Clear cell carcinoma, *EC*: Endometrioid carcinoma, *HGSC*: High grade serous carcinoma, *MC*: Mucinous carcinoma

Table 10 Predictive classification statistics for binary XGB models using PGs

Cohort	Histotype	Sensitivity	Specificity	AUC	Features
GSE6008	CCC	0.93	1	1	17
GSE6008	EC	0.57	0.82	0.75	10
GSE6008	HGSC	0.88	0.8	0.9	16
GSE6008	MC	0.79	0.93	0.93	25
GSE44104	CCC	0.65	0.96	0.94	21
GSE44104	EC	0.5	0.91	0.85	10
GSE44104	HGSC	0.86	0.86	0.9	16
GSE44104	MC	0.99	1	1	5
E-MTAB-1814	CCC	0.79	0.95	0.87	30
E-MTAB-1814	EC	0.64	0.88	0.86	5
E-MTAB-1814	HGSC	0.7	0.89	0.9	9
E-MTAB-1814	MC	0.55	0.82	0.73	11
GSE2109	CCC	0.61	0.88	0.84	25
GSE2109	EC	0.59	0.75	0.72	10
GSE2109	HGSC	0.76	0.81	0.86	16
GSE2109	MC	0.74	0.91	0.91	27

Mean values for cross-validated XGB classification (folds=5, resamples=100) constructed through PGs. *Sensitivity*: True positives/(True positives + False negatives), *Specificity*: True negatives/(True negatives + False positives)), *AUC*: Area under ROC curve, *Features*: Genes used as predictors in classifier model, *PG*: Predictor Gene (classified as significantly differentially expressed gene in 2/3 possible histotype comparisons for a histotype of interest used as reference in comparisons). *CCC*: Clear cell carcinoma, *EC*: Endometrioid carcinoma, *HGSC*: High grade serous carcinoma, *MC*: Mucinous carcinoma

Table 11 Predictive classification statistics for binary XGB models using HSGs

Cohort	Histotype	Sensitivity	Specificity	AUC	Features
GSE6008	CCC	0.92	1	1	6
GSE6008	EC	NA	NA	NA	NA
GSE6008	HGSC	0.97	0.87	0.81	11
GSE6008	MC	0.78	0.96	0.91	16
GSE44104	CCC	0.67	0.81	0.76	6
GSE44104	EC	NA	NA	NA	NA
GSE44104	HGSC	0.88	0.82	0.93	6
GSE44104	MC	1	1	1	26
E-MTAB-1814	CCC	0.78	0.92	0.86	6
E-MTAB-1814	EC	NA	NA	NA	NA
E-MTAB-1814	HGSC	0.78	0.82	0.86	NA
E-MTAB-1814	MC	0.75	0.87	0.86	9
GSE2109	CCC	0.7	0.96	0.88	8
GSE2109	EC	NA	NA	NA	NA
GSE2109	HGSC	0.9	0.77	0.9	7
GSE2109	MC	0.72	0.96	0.97	11

Mean values for cross-validated XGB classification (folds=5, resamples=100) constructed through PGs. *Sensitivity*: True positives/(True positives + False negatives), *Specificity*: True negatives/(True negatives + False positives)), *AUC*: Area under ROC curve, *Features*: Genes used as predictors in classifier model, *HSG*: Histotype specific gene (classified as significantly differentially expressed gene in 3/3 possible histotype comparisons for a histotype of interest used as reference in comparisons), *CCC*: Clear cell carcinoma, *EC*: Endometrioid carcinoma, *HGSC*: High grade serous carcinoma, *MC*: Mucinous carcinoma

Figure 5 PCA plots for 116 probes mapping to HSGs and top 116 most variable probes in test-cohort dataset GSE6008

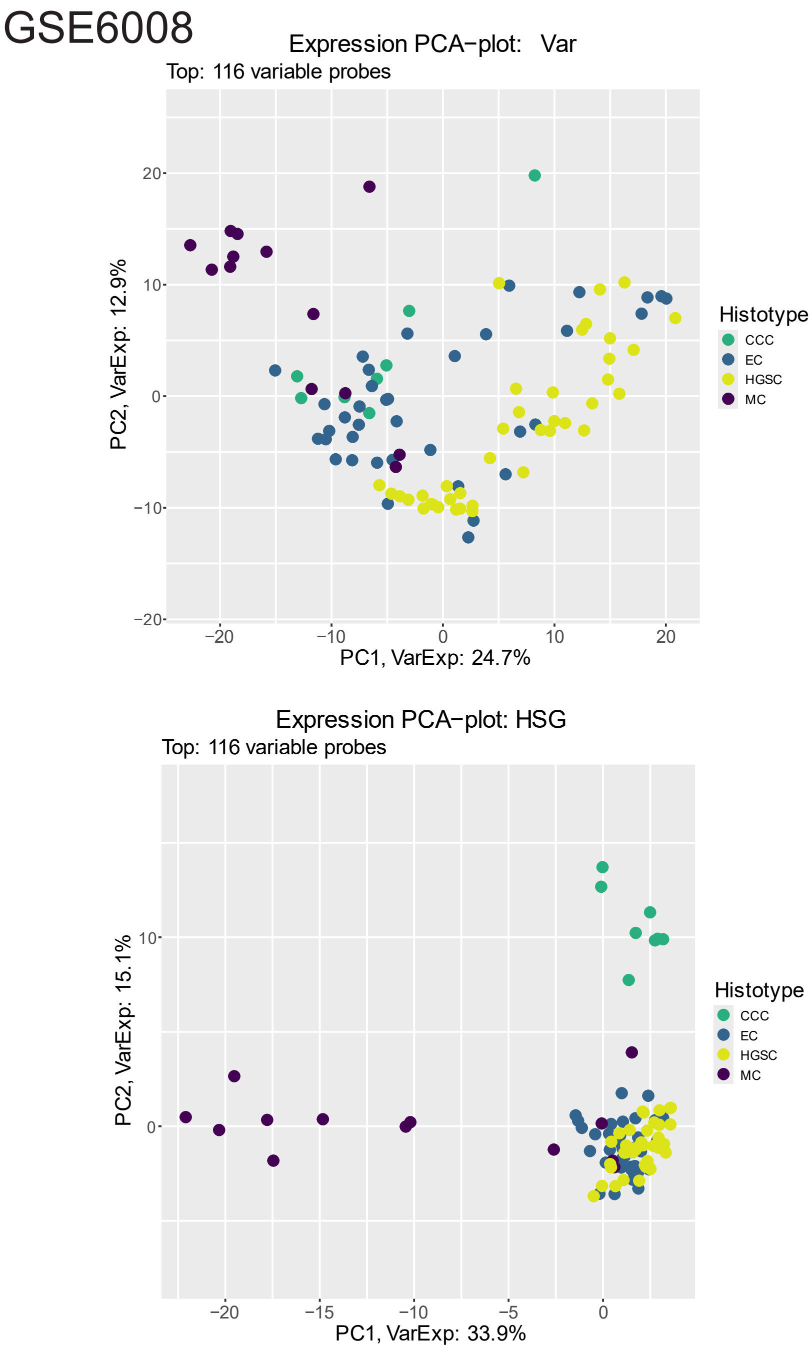

HSG: Histotype specific gene (classified as significantly differentially expressed gene in 3/3 possible histotype comparisons for a histotype of interest used as reference in comparisons), *CCC*: Clear cell carcinoma, *EC*: Endometrioid carcinoma, *HGSC*: High grade serous carcinoma, *MC*: Mucinous carcinoma. Created in r-package "ggplot"

Figure 6 PCA plots for 287 probes mapping to HSGs and top 287 most variable probes in test-cohort dataset GSE44104

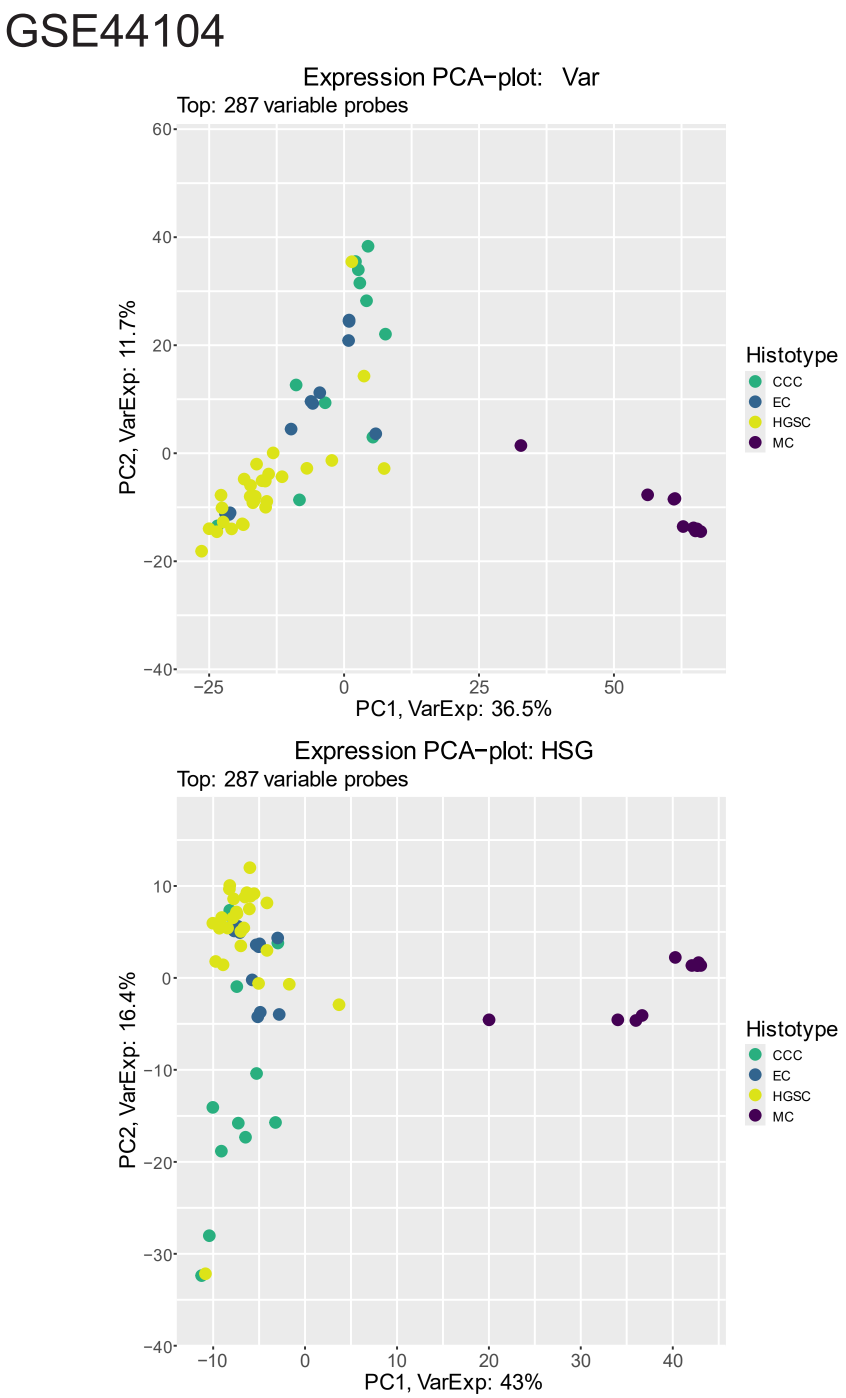

HSG: Histotype specific gene (classified as significantly differentially expressed gene in 3/3 possible histotype comparisons for a histotype of interest used as reference in comparisons), *CCC*: Clear cell carcinoma, *EC*: Endometrioid carcinoma, *HGSC*: High grade serous carcinoma, *MC*: Mucinous carcinoma. Created in r-package "ggplot"

Figure 7 PCA plots for 279 probes mapping to HSGs and top 279 most variable probes in test-cohort dataset E-MTAB-1814

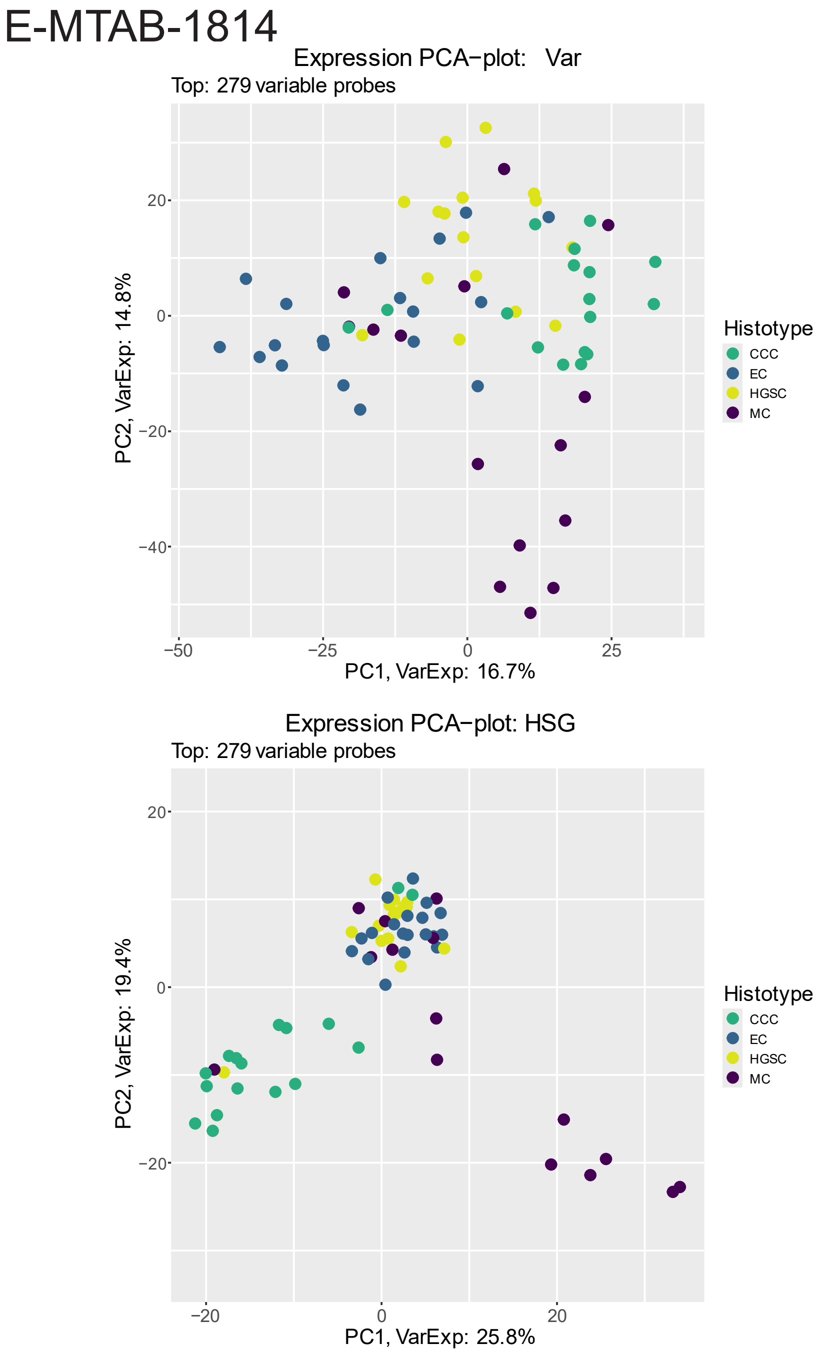

HSG: Histotype specific gene (classified as significantly differentially expressed gene in 3/3 possible histotype comparisons for a histotype of interest used as reference in comparisons), *CCC*: Clear cell carcinoma, *EC*: Endometrioid carcinoma, *HGSC*: High grade serous carcinoma, *MC*: Mucinous carcinoma. Created in r-package "ggplot"

Figure 8 PCA plots for 371 probes mapping to HSGs and top 371 most variable probes in test-cohort dataset GSE2109

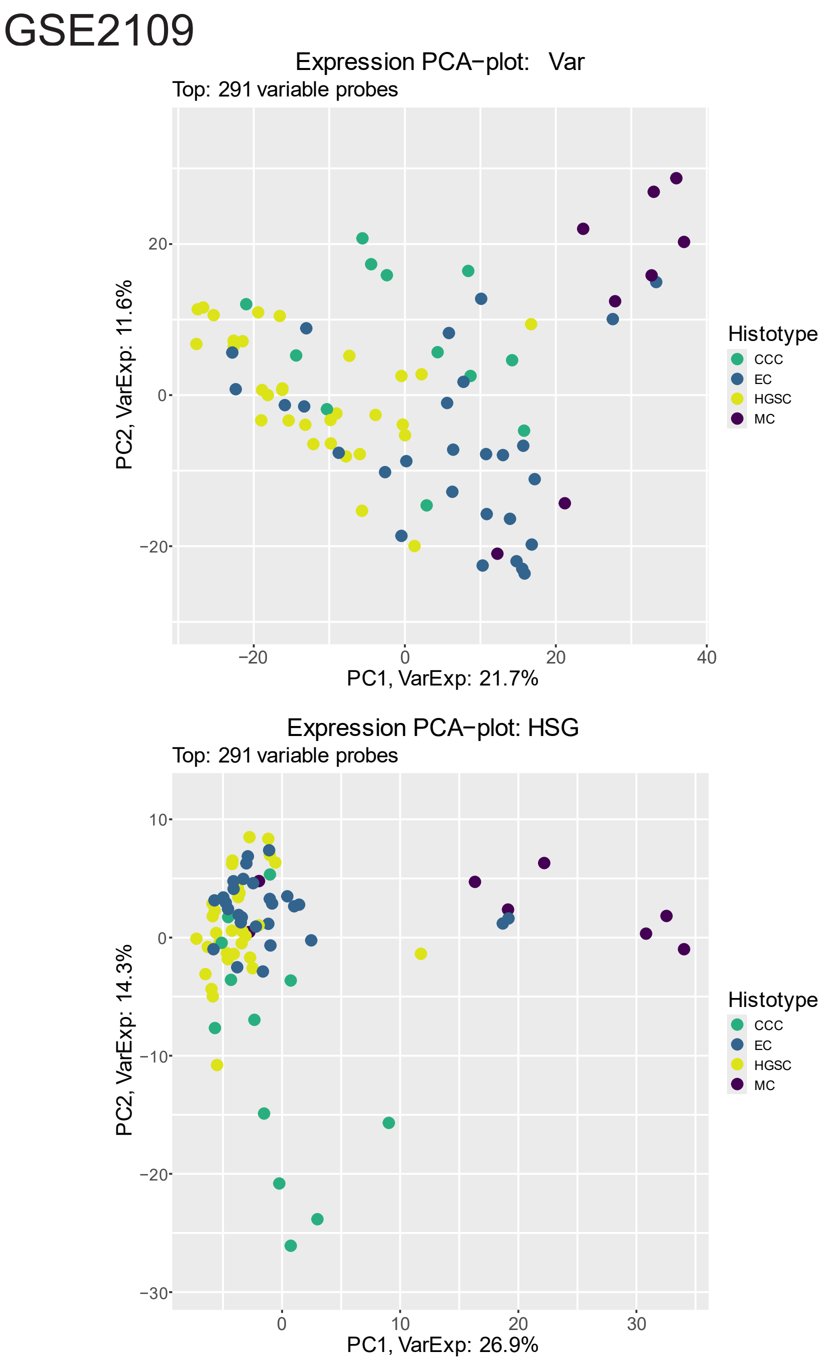

HSG: Histotype specific gene (classified as significantly differentially expressed gene in 3/3 possible histotype comparisons for a histotype of interest used as reference in comparisons), *CCC*: Clear cell carcinoma, *EC*: Endometrioid carcinoma, *HGSC*: High grade serous carcinoma, *MC*: Mucinous carcinoma. Created in r-package "ggplot"

Figure 9 Hierarchical clustering of training cohort

Training Cohort

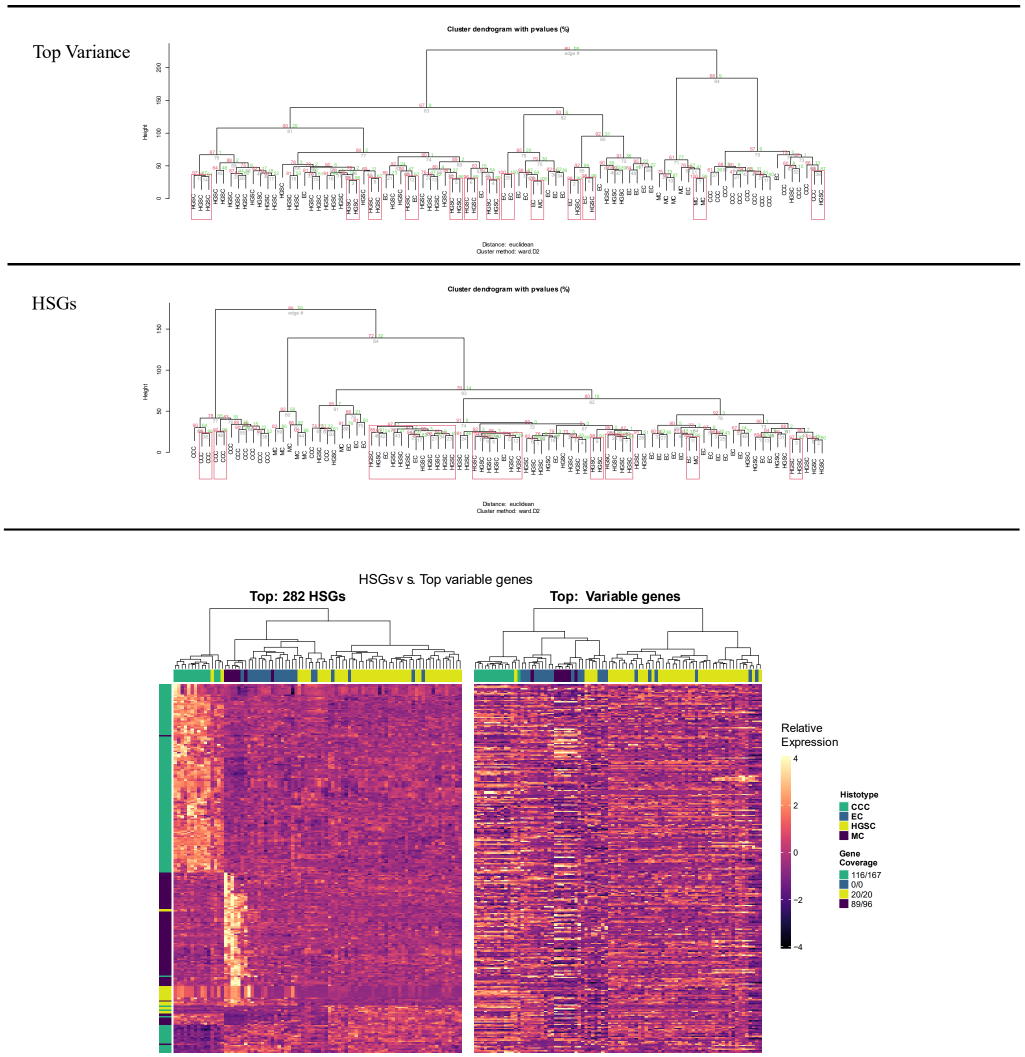

Upper plots: Hierarchical clustering of training cohort (Euclidean distance, Ward.D2) with p-values, bootstrap probability for training cohort for 282 HSGs (bottom) and the 282 most variable genes (top). *Top Variance/variable*: Most n (n=number of HSGs in dataset) variable genes in cohort, *Red AU*: approximately unbiased p-value, *Green BP*: bootstrap probability. *Red box*: AU >= 0.95 (95%). Created in r package "pvclust". Lower plot: Heatmaps of training cohort dataset with hierarchical clustering (Euclidean distance, Ward.D2) for HSGs (left) and the most variable genes (right). Color mapping for heatmaps indicative of z-score for gene expression. *HSG:* Histotype specific gene (classified as significantly differentially expressed gene in 3/3 possible histotype comparisons for a histotype of interest used as reference in comparisons), *CCC*: Clear cell carcinoma, *EC*: Endometroid carcinoma, *HGSC*: High-grade serous carcinoma, *MC*: Mucinous carcinoma, *Gene Coverage*: Number of HSGs with a mapping probe in the array, in relation to total number of HSGs with gene symbol. Created in r-package "ComplexHeatmap"

Figure 10 Hierarchical clustering of test cohort dataset GSE6008

GSE6008

Top Variance

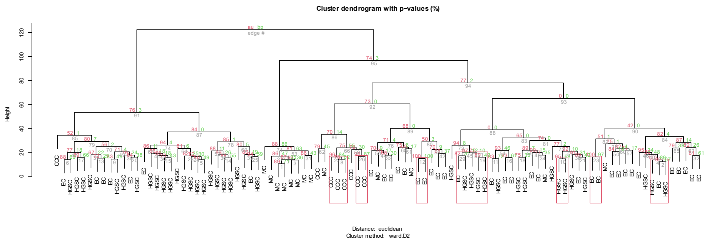

HSGs

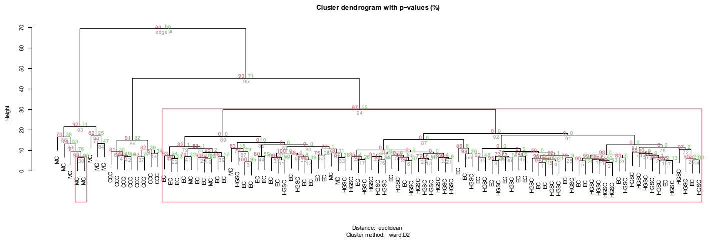

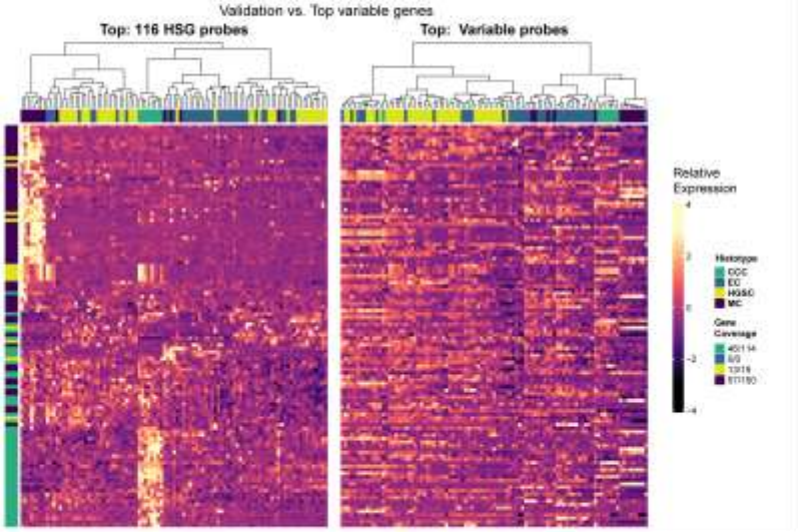

Upper plots: Hierarchical clustering of test cohort dataset GSE6008 (Euclidean distance, Ward.D2) with p-values, bootstrap probability for 116 probes mapping to HSGs (bottom) and the 116 most variable probes (top). *Top Variance/variable*: Most n (n=number of HSGs in dataset) variable genes in cohort, Red AU: approximately unbiased p-value, Green BP: bootstrap probability. Red box: AU >= 0.95 (95%). Created in r package "pvclust". Lower plot: Heatmaps of training cohort dataset with hierarchical clustering (Euclidean distance, Ward.D2) for HSGs (left) and the most variable genes (right). Color mapping for heatmaps indicative of z-score for gene expression. *HSG:* Histotype specific gene (classified as significantly differentially expressed gene in 3/3 possible histotype comparisons for a histotype of interest used as reference in comparisons), *CCC*: Clear cell carcinoma, *EC*: Endometroid carcinoma, *HGSC*: High-grade serous carcinoma, *MC*: Mucinous carcinoma, *Gene Coverage*: Number of HSGs with a mapping probe in the array, in relation to total number of HSGs with gene symbol. Created in r-package "ComplexHeatmap"

Figure 11 *Upper plots: Hierarchical clustering of test cohort dataset GSE44104*

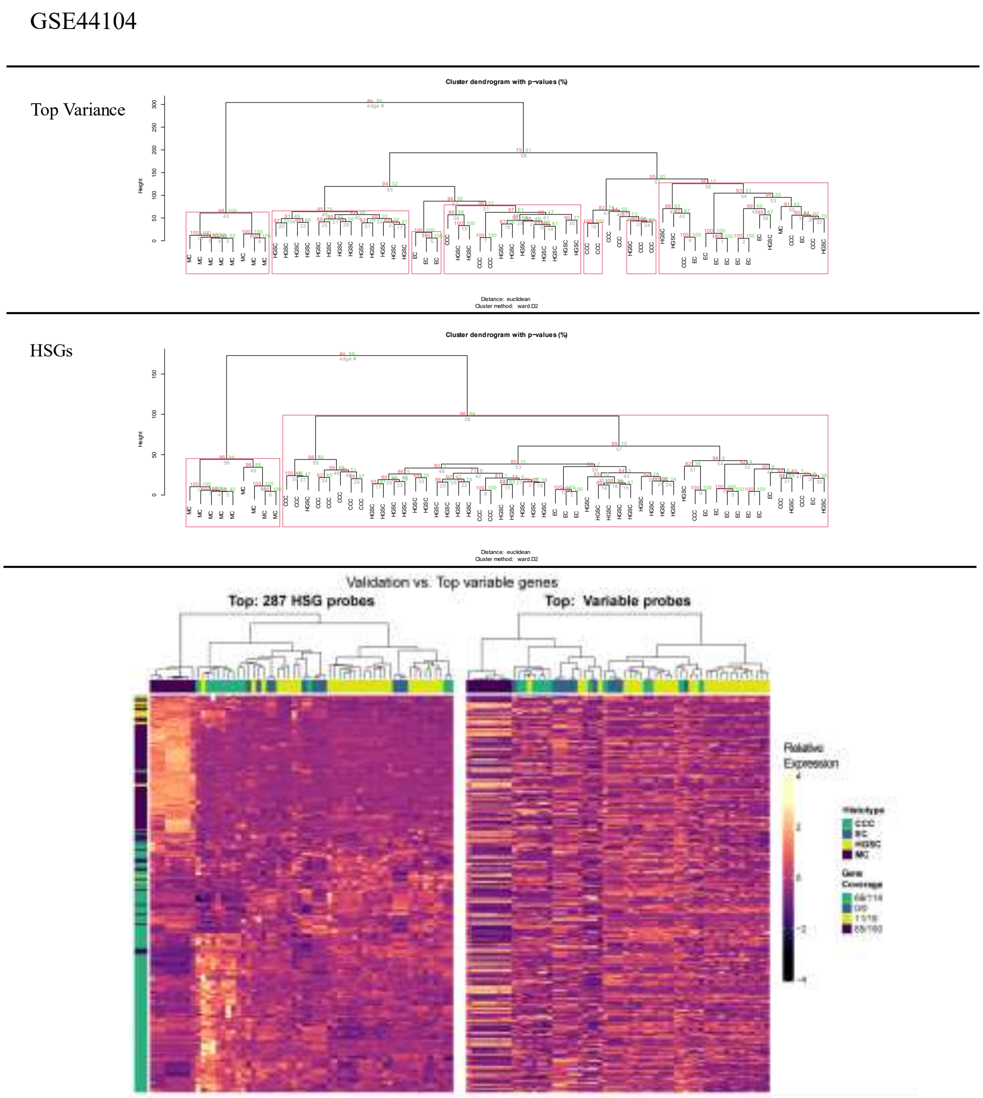

Upper plots: Hierarchical clustering of test cohort dataset GSE44104 (Euclidean distance, Ward.D2) with p-values, bootstrap probability for 287 probes mapping to HSGs (bottom) and the 287 most variable probes (top). *Top Variance/variable*: Most n (n=number of HSGs in dataset) variable genes in cohort, *Red AU*: approximately unbiased p-value, *Green BP*: bootstrap probability. *Red box*: AU >= 0.95 (95%). Created in r package "pvclust". Lower plot: Heatmaps of training cohort dataset with hierarchical clustering (Euclidean distance, Ward.D2) for HSGs (left) and the most variable genes (right). Color mapping for heatmaps indicative of z-score for gene expression. *HSG:* Histotype specific gene (classified as significantly differentially expressed gene in 3/3 possible histotype comparisons for a histotype of interest used as reference in comparisons), *CCC*: Clear cell carcinoma, *EC*: Endometroid carcinoma, *HGSC*: High-grade serous carcinoma, *MC*: Mucinous carcinoma, *Gene Coverage*: Number of HSGs with a mapping probe in the array, in relation to total number of HSGs with gene symbol. Created in r-package "ComplexHeatmap"

Figure 12 Hierarchical clustering of test cohort dataset GSE2109

GSE2109

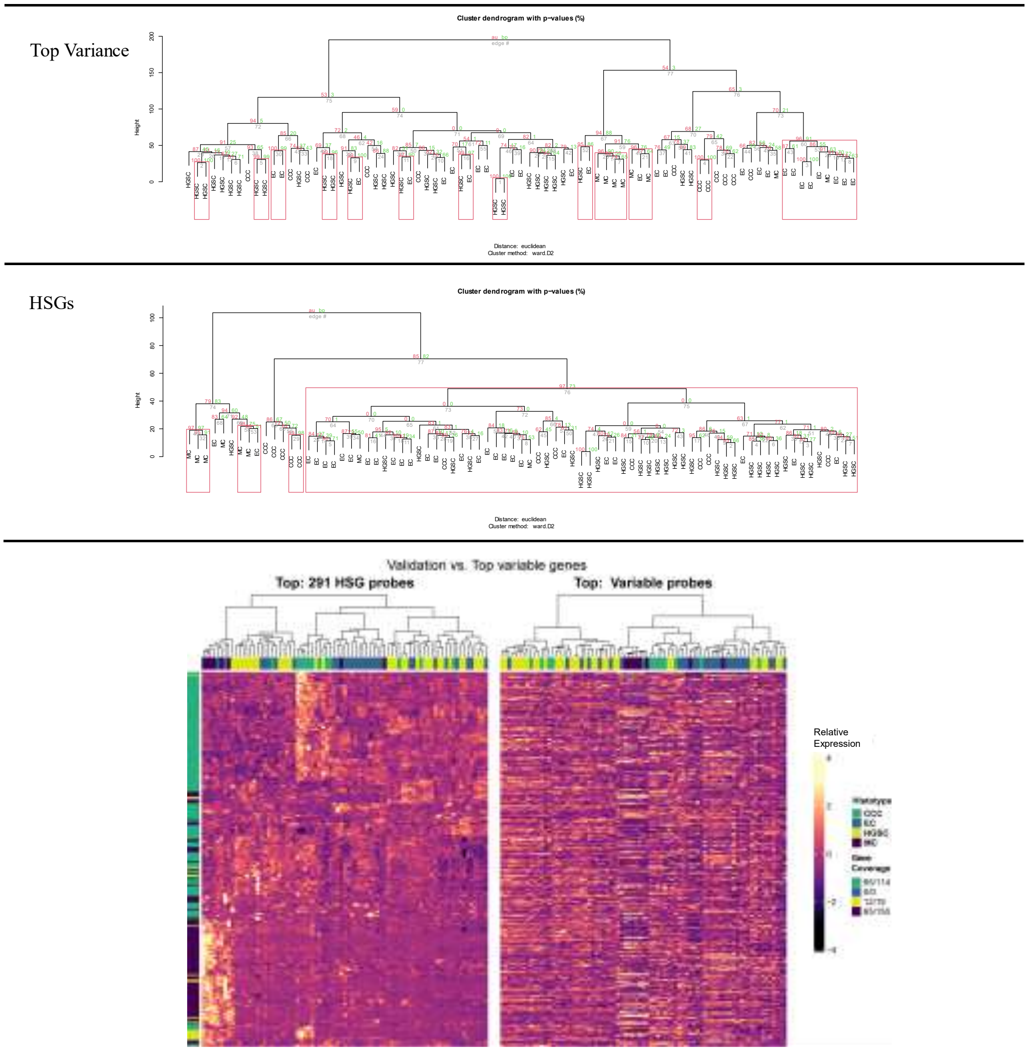

Upper plots: Hierarchical clustering of test cohort dataset GSE2109 (Euclidean distance, Ward.D2) with p-values, bootstrap probability for 371 probes mapping to HSGs (bottom) and the 371 most variable probes (top). *Top Variance/variable*: Most n (n=number of HSGs in dataset) variable genes in cohort, Red AU: approximately unbiased p-value, Green BP: bootstrap probability. Red box: AU >= 0.95 (95%). Created in r package “pvclust”. Lower plot: Heatmaps of training cohort dataset with hierarchical clustering (Euclidean distance, Ward.D2) for HSGs (left) and the most variable genes (right). Color mapping for heatmaps indicative of z-score for gene expression. *HSG:* Histotype specific gene (classified as significantly differentially expressed gene in 3/3 possible histotype comparisons for a histotype of interest used as reference in comparisons), *CCC*: Clear cell carcinoma, *EC*: Endometroid carcinoma, *HGSC*: High-grade serous carcinoma, *MC*: Mucinous carcinoma, *Gene Coverage*: Number of HSGs with a mapping probe in the array, in relation to total number of HSGs with gene symbol. Created in r-package “ComplexHeatmap”

Figure 13 Hierarchical clustering of test cohort dataset E-MTAB-1814

EMTAB1814

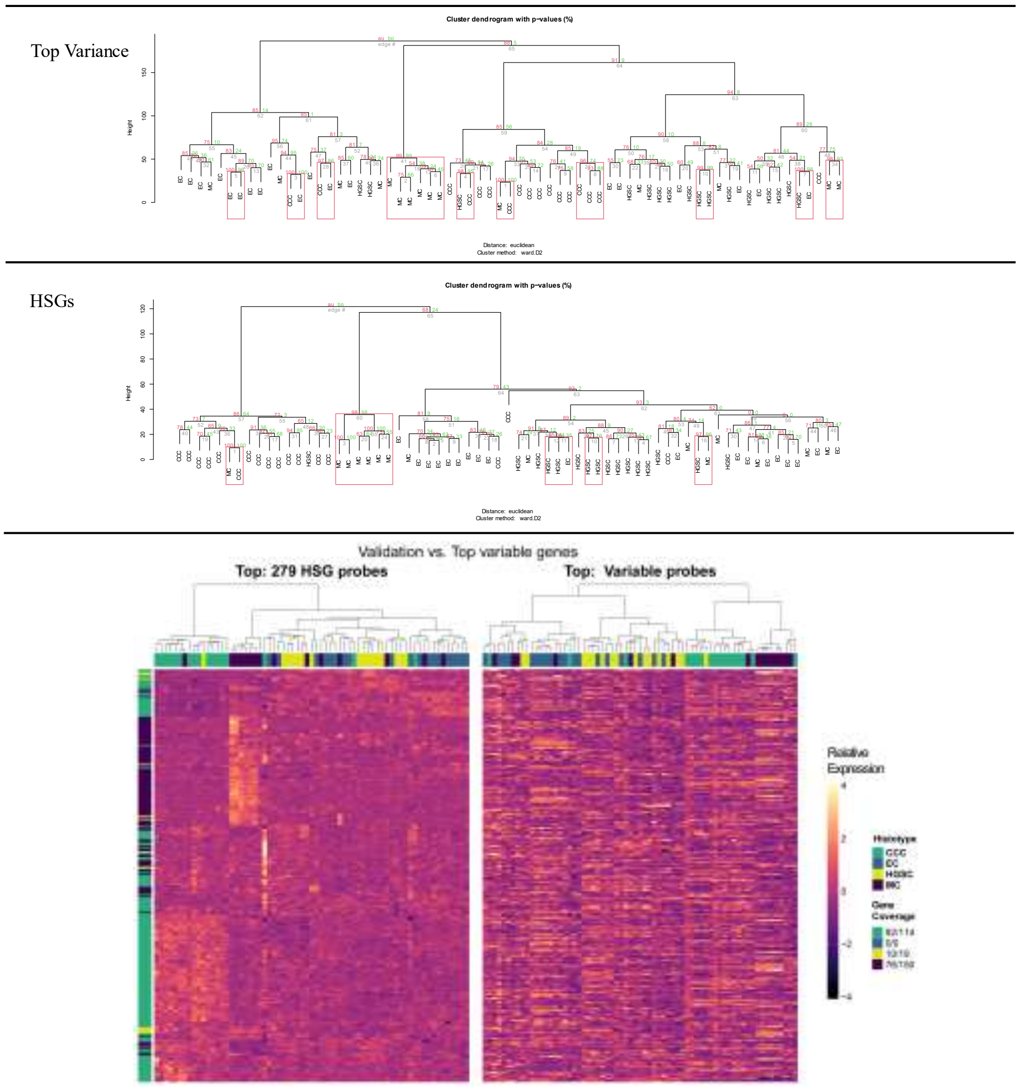

Upper plots: Hierarchical clustering of test cohort dataset E-MTAB-1814 (Euclidean distance, Ward.D2) with p-values, bootstrap probability for 279 probes mapping to HSGs (bottom) and top 279 most variable probes (top). *Top Variance/variable*: Most n (n=number of HSGs in dataset) variable genes in cohort, *Red AU*: approximately unbiased p-value, *Green BP*: bootstrap probability. *Red box*: AU >= 0.95 (95%). Created in r package "pvclust". Lower plot: Heatmaps of training cohort dataset with hierarchical clustering (Euclidean distance, Ward.D2) for HSGs (left) and the most variable genes (right). Color mapping for heatmaps indicative of z-score for gene expression. *HSG:* Histotype specific gene (classified as significantly differentially expressed gene in 3/3 possible histotype comparisons for a histotype of interest used as reference in comparisons), *CCC*: Clear cell carcinoma, *EC*: Endometroid carcinoma, *HGSC*: High-grade serous carcinoma, *MC*: Mucinous carcinoma, *Gene Coverage*: Number of HSGs with a mapping probe in the array, in relation to total number of HSGs with gene symbol. Created in r-package "ComplexHeatmap"

Table 12 Deg results for training and test cohorts

	Training		Test							
			GSE6008		GSE44104		GSE2019		E-MTAB-1814	
**Comparison**	**Down**	**Up**	**Down**	**Up**	**Down**	**Up**	**Down**	**Up**	**Down**	**Up**
CCC_EC	325	406	203	395	84	46	52	45	565	529
CCC_HGSC	611	698	364	486	199	342	52	57	450	497
CCC_MC	384	442	259	317	2257	1410	303	46	105	217
EC_CCC	405	328	395	203	46	84	45	52	529	565
EC_HGSC	19	100	60	43	369	579	80	124	62	180
EC_MC	119	43	282	81	2292	2027	244	13	8	13
HGSC_CCC	698	616	486	364	342	199	57	52	497	450
HGSC_EC	98	32	43	60	579	369	124	80	180	62
HGSC_MC	445	327	421	283	2866	2166	1031	363	216	164
MC_CCC	407	390	317	259	1410	2257	46	303	217	105
MC_EC	29	119	81	282	2027	2292	13	244	13	8
MC_HGSC	300	445	283	421	2166	2866	363	1031	164	216

Significant differentially expressed gene (DEGs; Training) and probes (test) calls for training and test cohort (Training: DESeq2 fdr < 0.05 | Test: Limma, Benjamini Hochberg adjusted p-value < 0.05) for case (histotype in the comparison to the left) when compared to control (histotype in the comparison to the right). Comparisons for a dataset with less than 200 total significant differentially expressed genes/probes are highlighted in light grey. *Up*: Upregulated DEGs, *Down*: Downregulated DEGs, *CCC*: Clear cell carcinoma, *EC*: Endometroid carcinoma, *HGSC*: High-grade serous carcinoma, *MC*: Mucinous carcinoma

Table 13 Statistics for CCC HSGs defined as DEG in >=2/4 cohorts and >33% of all possible histotype comparisons with associated histotype used as reference

Gene	Total DEG frequency (%)	GSE6008	GSE44104	E-MTAB-1814	GSE2109
GABARAPL1	0.83	3	3	2	2
ARID3A	0.67	3	2	3	NA
CTH	0.67	3	2	3	NA
GPX3	0.67	3	1	3	1
LAMC1	0.67	3	3	2	NA
RNASET2	0.67	3	2	3	NA
STC1	0.67	3	2	1	2
WARS1	0.67	3	1	2	2
NABP1	0.67	3	2	2	NA
PGBD5	0.67	3	1	3	NA
VCAN	0.67	3	1	3	NA
BATF3	0.5	3	1	2	NA
CLIC5	0.5	3	1	2	NA
CSGALNACT1	0.5	3	2	1	NA
ELAPOR1	0.5	2	1	1	2
IGFBP3	0.5	3	1	1	1
IL6	0.5	3	NA	3	NA
NFIA	0.5	NA	2	3	1
NUP50	0.5	3	1	2	NA
PTH1R	0.5	3	NA	3	NA
PTHLH	0.5	3	NA	3	NA
RIMKLB	0.5	NA	3	3	NA
TRABD2B	0.5	NA	2	3	1
WDR91	0.5	3	1	2	NA
POU2AF3	0.42	NA	1	3	1
RDH10	0.42	NA	1	3	1
SLC7A2	0.42	3	NA	2	NA
SYNE2	0.42	3	1	1	NA
CEP44	0.33	NA	1	3	NA
CR1L	0.33	NA	2	2	NA
CYTIP	0.33	3	NA	1	NA
GALNT11	0.33	3	1	NA	NA

KIFC3	0.33	2	1	1	NA
KNG1	0.33	2	NA	2	NA
PLEKHS1	0.33	NA	2	2	NA
SLC35F5	0.33	NA	2	2	NA
SLC5A1	0.33	NA	1	2	1
SLC8A1	0.33	NA	2	1	1

HSG: Histotype specific gene (classified as significantly differentially expressed gene in 3/3 possible histotype comparisons for a histotype of interest used as reference in comparisons), *DEG frequency*: Significant differentially expressed gene calls (fdr < 0.05, absolute value log2FoldChange > 0.585) divided by total number of possible significant DEG calls, *CCC*: Clear cell carcinoma. Value in cells under test cohort name indicative of the number of significant DEG calls made for possible (n=3) histotype comparisons using histotype associated with HSG as reference

Table 14 Statistics for HGSC HSGs defined as DEG in >=2/4 cohorts and >33% of all possible histotype comparisons with associated histotype used as reference

Gene	Total DEG frequency (%)	GSE6008	GSE44104	E-MTAB-1814	GSE2109
RASSF6	0.5	NA	3	NA	3
UGT1A1	0.5	2	1	2	1
UGT1A10	0.5	2	1	2	1
UGT1A3	0.5	2	1	2	1
UGT1A6	0.5	2	1	2	1
UGT1A9	0.5	2	1	2	1
MUC13	0.42	2	2	NA	1
TGM1	0.42	2	2	1	NA
UGT1A8	0.42	1	1	2	1
AKR1B10	0.33	2	1	NA	1
CTCFL	0.33	NA	2	NA	2
UGT1A4	0.33	NA	1	2	1
UGT1A5	0.33	NA	1	2	1
UGT1A7	0.33	NA	1	2	1

HSG: Histotype specific gene (classified as significantly differentially expressed gene in 3/3 possible histotype comparisons for a histotype of interest used as reference in comparisons), *DEG frequency*: Significant differentially expressed gene calls (fdr < 0.05, absolute value log2FoldChange > 0.585) divided by total number of possible significant DEG calls, *HGSC*: High-grade serous carcinoma. Value in cells under test cohort name indicative of the number of significant DEG calls made for possible (n=3) histotype comparisons using histotype associated with HSG as reference

Table 15 Statistics for MC HSGs defined as DEG in >=2/4 cohorts and >33% of all possible histotype comparisons with associated histotype used as reference

Gene	Total DEG frequency (%)	GSE6008	GSE44104	EMTAB1814	GSE2109
ATP10B	0.83	3	3	1	3
VILL	0.83	3	3	1	3
AKR7A3	0.75	3	3	NA	3
BCL2L14	0.75	3	3	NA	3
CLDN18	0.75	2	3	1	3
CYP3A5	0.75	3	3	1	2
GPX2	0.75	2	3	1	3
AHCYL2	0.67	2	3	NA	3
ANXA10	0.67	2	3	NA	3
CES2	0.67	3	3	NA	2
FMO5	0.67	2	3	NA	3
GLDC	0.67	2	3	2	1
KRT20	0.67	2	3	1	2
LYZ	0.67	3	3	NA	2
MUC3A	0.67	2	3	1	2
NR1I2	0.67	2	3	NA	3
VIL1	0.67	2	3	NA	3
A1CF	0.58	2	3	NA	2
BTNL8	0.58	2	3	NA	2
CDHR2	0.58	2	3	NA	2
CDHR5	0.58	2	3	NA	2
CTSE	0.58	2	3	NA	2
RAPGEFL1	0.58	2	3	NA	2
TM4SF4	0.58	2	3	NA	2
ADH1C	0.5	2	3	NA	1
AGMAT	0.5	1	3	NA	2
ALDOB	0.5	2	3	NA	1
ANXA13	0.5	2	3	NA	1
CYP3A4	0.5	2	3	NA	1
GALNT5	0.5	NA	3	NA	3
LRRC66	0.5	NA	3	NA	3
MAL	0.5	2	3	NA	1

NHERF4	0.5	NA	3	1	2
SLC26A3	0.5	2	3	NA	1
ADTRP	0.42	NA	3	NA	2
CLDN23	0.42	NA	3	NA	2
GAU1	0.42	NA	3	NA	2
MMP1	0.42	2	3	NA	NA
OTC	0.42	2	3	NA	NA
SLC39A5	0.42	NA	3	NA	2
SULT1B1	0.42	NA	3	NA	2
TNNT1	0.42	2	2	NA	1
TUBAL3	0.42	NA	3	1	1
VSIG1	0.42	NA	3	NA	2
ANKRD33B	0.33	NA	3	NA	1
CYSTM1	0.33	NA	3	NA	1
LIPH	0.33	NA	3	NA	1
MOGAT2	0.33	NA	3	NA	1
NAT2	0.33	NA	3	NA	1
PRIMA1	0.33	NA	3	NA	1

HSG: Histotype specific gene (classified as significantly differentially expressed gene in 3/3 possible histotype comparisons for a histotype of interest used as reference in comparisons), *DEG frequency*: Significant differentially expressed gene calls (fdr < 0.05, absolute value log2FoldChange > 0.585) divided by total number of possible significant DEG calls, M*C*: Mucinous carcinoma. Value in cells under test cohort name indicative of the number of significant DEG calls made for possible (n=3) histotype comparisons using histotype associated with HSG as reference

Table 16 Statistics for HSGs found in multiclass predictive models and defined as DEG in >=2/4 external cohorts for its associated histotype

Gene	Histotype	Total DEG frequency (%)	GSE6008	GSE2109	GSE44104	E-MTAB-1814
ARID3A	CCC	0.67	3	NA	2	3
RNASET2	CCC	0.67	3	NA	2	3
WARS1	CCC	0.67	3	2	1	2
VCAN	CCC	0.58	3	NA	1	3
IGFBP3	CCC	0.5	3	1	1	1
RIMKLB	CCC	0.5	NA	NA	3	3
RDH10	CCC	0.42	NA	1	1	3
SYNE2	CCC	0.42	3	NA	1	1
CEP44	CCC	0.33	NA	NA	1	3
SLC35F5	CCC	0.33	NA	NA	2	2
RASSF6	HGSC	0.5	NA	3	3	NA
UGT1A6	HGSC	0.5	2	1	1	2
MUC13	HGSC	0.42	2	1	2	NA
TGM1	HSGC	0.42	2	NA	2	1
UGT1A8	HSGC	0.42	1	1	1	2
AKR1B10	HGSC	0.33	2	1	1	NA
CTCFL	HGSC	0.33	NA	2	2	NA
KRT20	MC	0.58	2	2	3	1
CDHR2	MC	0.58	2	2	3	NA
CDHR5	MC	0.58	2	2	3	NA
AGMAT	MC	0.5	1	2	3	NA
ALDOB	MC	0.5	2	1	3	NA
ADTRP	MC	0.42	NA	2	3	NA
ANKRD33B	MC	0.33	NA	1	3	NA
CYSTM1	MC	0.33	NA	1	3	NA
PRIMA1	MC	0.33	NA	1	3	NA

DEG frequency: Significant differentially expressed gene calls (fdr < 0.05, absolute value log2FoldChange > 0.585) divided by total number of possible significant DEG calls, *CCC*: Clear cell carcinoma, *EC*: Endometroid carcinoma, *HGSC*: High-grade serous carcinoma, *MC*: Mucinous carcinoma. Value in cells under test cohort name indicative of the number of significant DEG calls made for possible (n=3) histotype comparisons using histotype associated with HSG as reference

Table 17 The number of differentially methylated probes and regions with methylation-type in the training cohort

**Comparison group**	**DMP - Total**	**DMP - Hyper methylation**	**DMP - Hypo methylation**	**DMR - Total**	**DMR- Hyper methylation**	**DMR - Hypo methylation**	**Histotype Specific DMRs**
EC – CCC	4051	1456	2595	180	33	147	35
HGSC – CCC	32171	9276	22895	560	72	488	35
MC – CCC	18286	3173	15113	327	62	265	35
CCC – EC	4051	2595	1456	180	147	33	0
HGSC – EC	7497	2427	5070	108	13	95	0
MC – EC	327	142	185	131	47	84	0
CCC – HGSC	32171	22895	9276	560	488	72	31
EC – HGSC	7497	5070	2427	108	95	13	31
MC – HGSC	38350	12200	26150	772	446	326	31
CCC – MC	18286	15113	3173	327	265	62	49
EC – MC	327	185	142	131	84	47	49
HGSC – MC	38350	26150	12200	772	326	446	49

DMP: Differentially methylated probe, *DMR*: Differentially methylated region, *Hypermethylation*: $\delta\ \beta > 0.2$, *Hypomethylation*: $\delta\ \beta < -0.2$, *CCC*: Clear cell carcinoma, *EC*: Endometroid carcinoma, *HGSC*: High-grade serous carcinoma, *MC*: Mucinous carcinoma

Figure 14 Differentially methylated probes for histotype comparisons

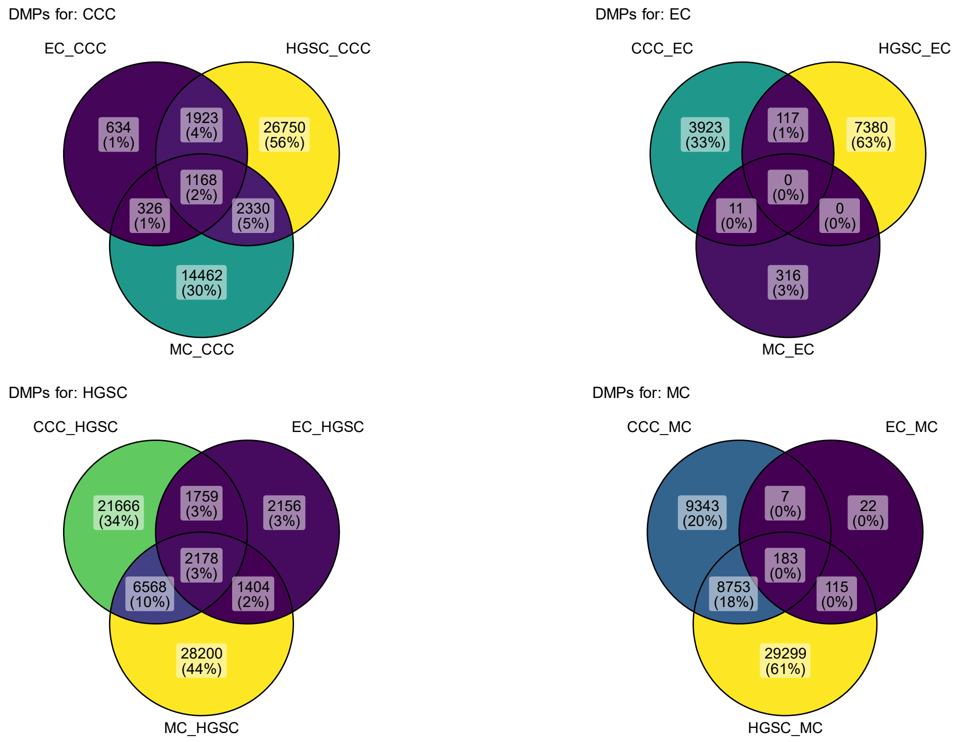

DMP: Differentially methylated probe, *CCC*: Clear cell carcinoma, *EC*: Endometroid carcinoma, *HGSC*: High-grade serous carcinoma, *MC*: Mucinous carcinoma. Created in r package “ggVennDiagram”

Figure 15 Differentially methylated regions for histotype comparisons

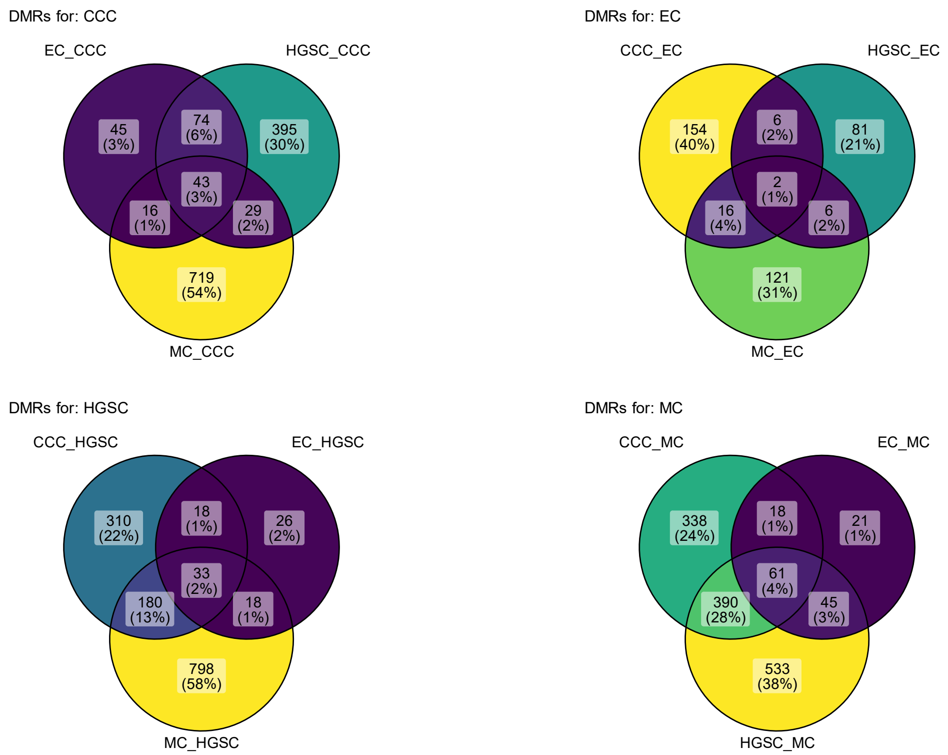

DMR: Differentially methylated region, *CCC*: Clear cell carcinoma, *EC*: Endometroid carcinoma, *HGSC*: High-grade serous carcinoma, *MC*: Mucinous carcinoma. Created in r package “ggVennDiagram”

Figure 16 Location of DMPs and DMP-types for comparisons involving histotype CCC

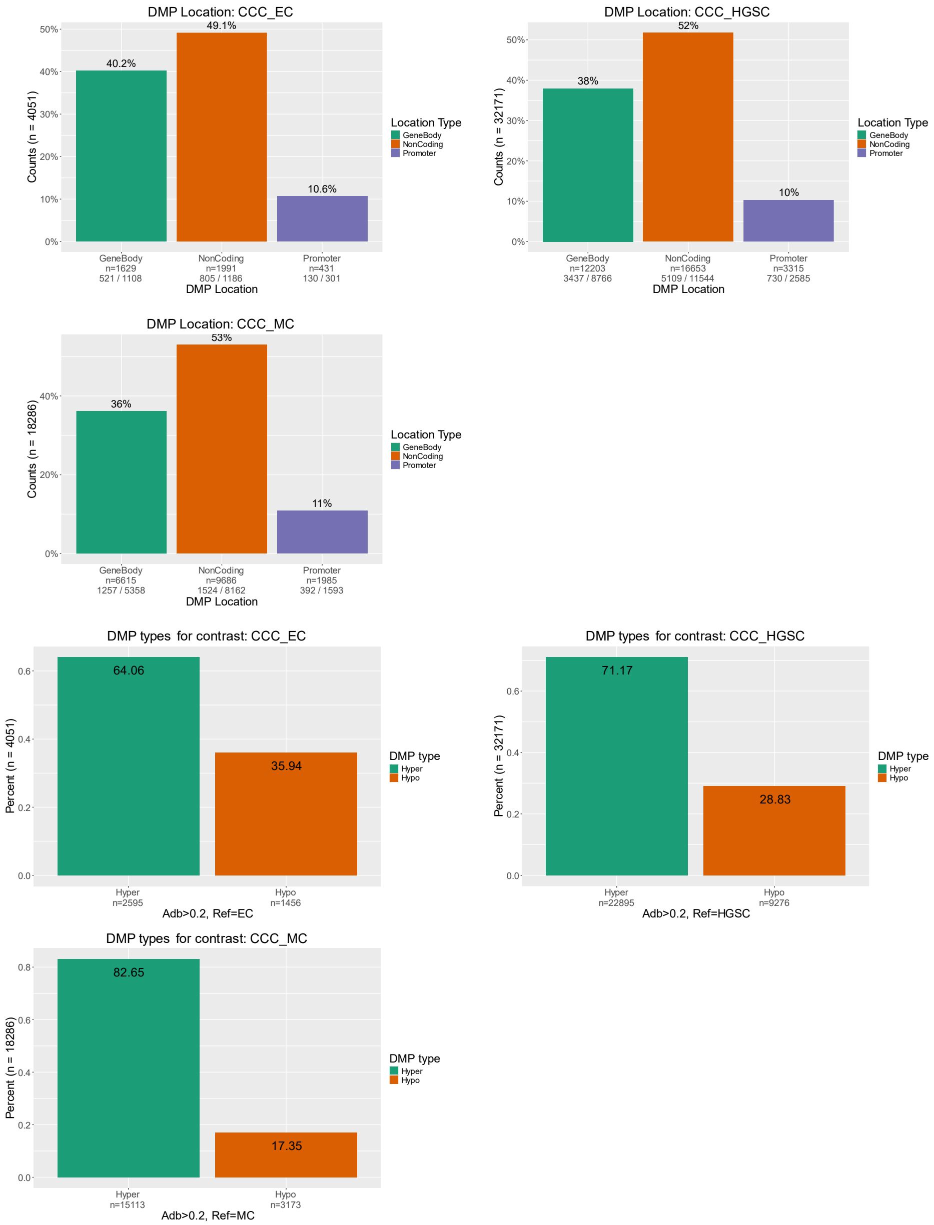

Upper Plots: DMP CpG site location on Hg38 genome for CCC. Numbers below location-type indicates total number of DMPs overlapping position (row 1) and Hypermethylated/Hypomethylated probes of the position-type (row 2). *Hyper*: Hypermethylated ($\delta\beta > 0.2$), *Hypo*: Hypomethylated ($\delta\beta < 0.2$), *Promoter*: 2000bp before TSS, 50bp after TSS. Lower plots: DMP methylation type for CCC, *Adb*: Absolute value of δ β, *DMP*: Differentially methylated probe, *CCC*: Clear cell carcinoma, *EC*: Endometroid carcinoma, *HGSC*: High-grade serous carcinoma, *MC*: Mucinous carcinoma. Created in R-package "ggplot"

Figure 17 Location of DMPs and DMP-types for comparisons involving histotype CCC

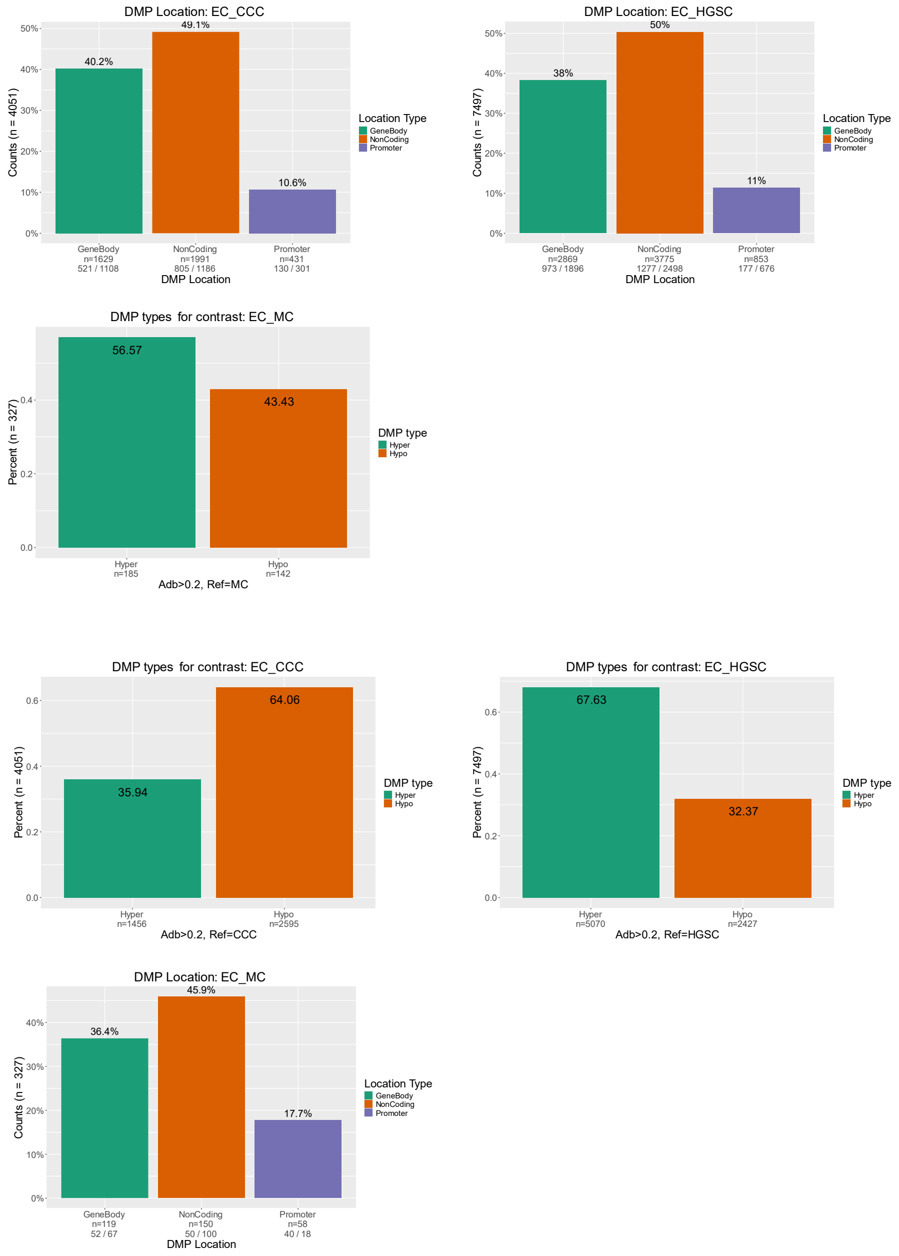

Upper Plots: Differentially methylated probes CpG site location on Hg38 genome for EC. Numbers below location-type indicates total number of DMPs overlapping position (row 1) and Hypermethylated/Hypomethylated probes of the position-type (row 2). *Hyper*: Hypermethylated (δβ > 0.2), *Hypo*: Hypomethylated (δβ < 0.2), *Promoter*: 2000bp before TSS, 50bp after TSS. Lower plots: DMP methylation type for CCC, *Adb*: Absolute value of δ β, *DMP*: Differentially methylated probe, *CCC*: Clear cell carcinoma, *EC*: Endometroid carcinoma, *HGSC*: High-grade serous carcinoma, *MC*: Mucinous carcinoma. Created in R-package "ggplot"

Figure 18 Location of DMPs and DMP-types for comparisons involving histotype HGSC

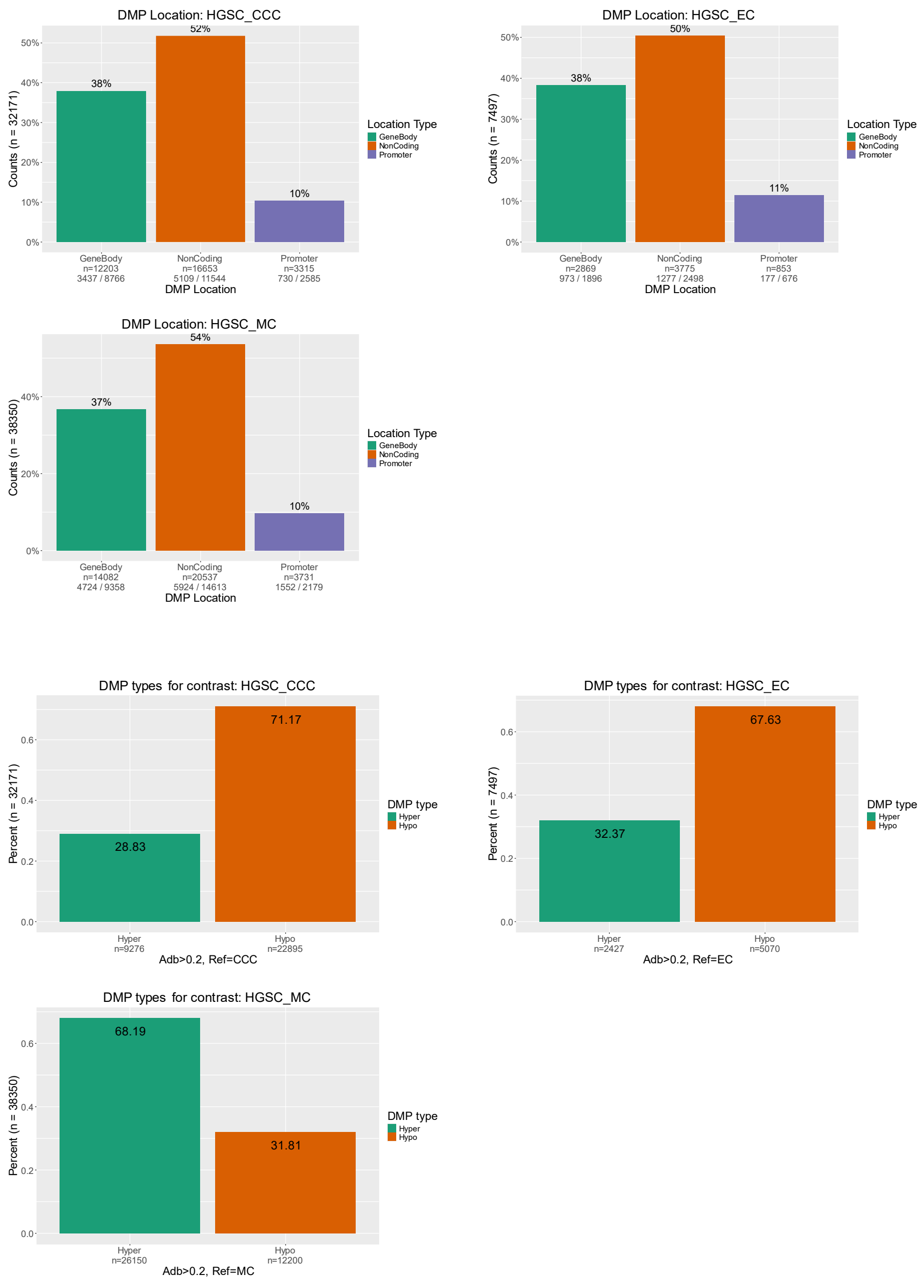

Upper Plots: Differentially methylated probes CpG site location on Hg38 genome for HGSC. Numbers below location-type indicates total number of DMPs overlapping position (row 1) and Hypermethylated/Hypomethylated probes of the position-type (row 2). *Hyper*: Hypermethylated (δβ > 0.2), *Hypo*: Hypomethylated (δβ < 0.2), *Promoter*: 2000bp before TSS, 50bp after TSS. Lower plots: DMP methylation type for CCC, *Adb*: Absolute value of δ β, *DMP*: Differentially methylated probe, *CCC*: Clear cell carcinoma, *EC*: Endometroid carcinoma, *HGSC*: High-grade serous carcinoma, *MC*: Mucinous carcinoma. Created in R-package "ggplot"

Figure 19 Location of DMPs and DMP-types for comparisons involving histotype MC

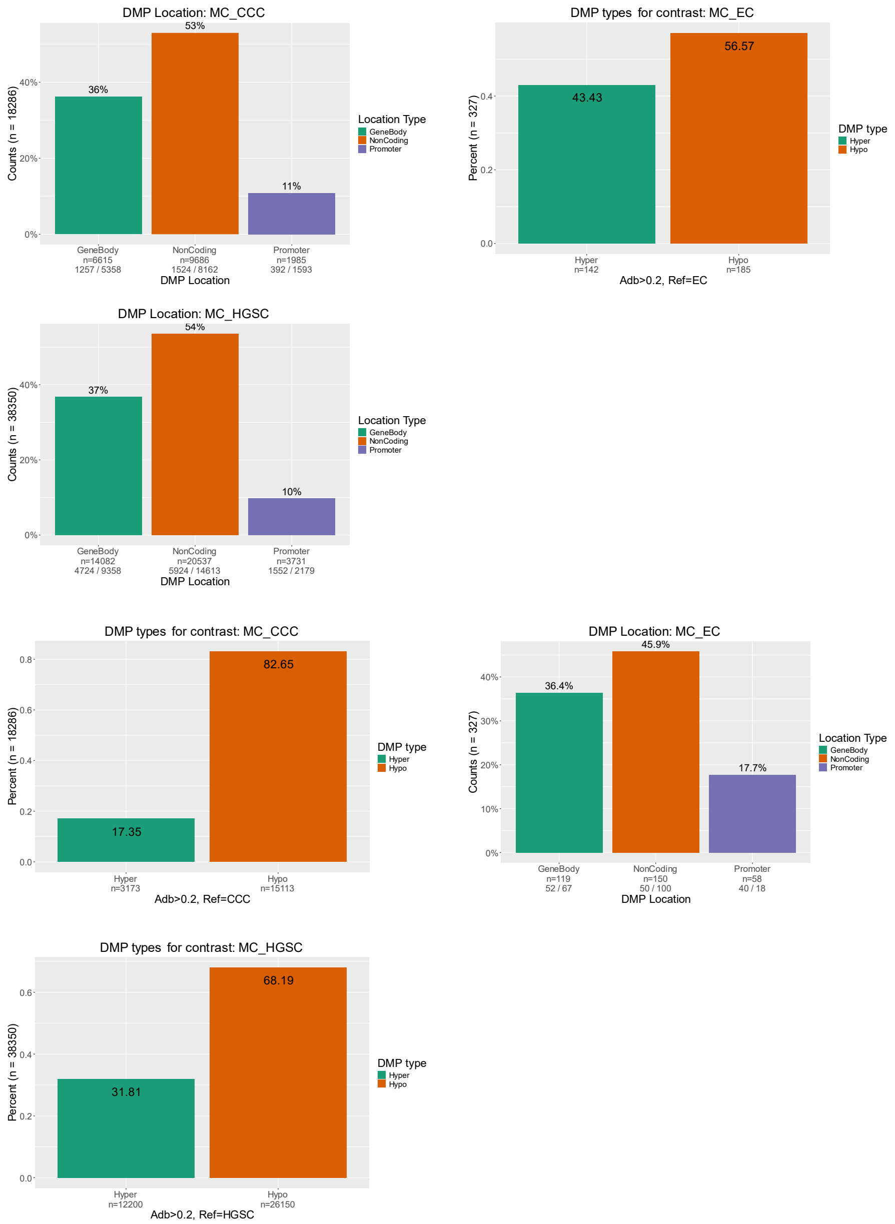

Upper Plots: Differentially methylated probes CpG site location on Hg38 genome for MC. Numbers below location-type indicates total number of DMPs overlapping position (row 1) and Hypermethylated/Hypomethylated probes of the position-type (row 2). *Hyper*: Hypermethylated ($\delta\beta > 0.2$), *Hypo*: Hypomethylated ($\delta\beta < 0.2$), *Promoter*: 2000bp before TSS, 50bp after TSS. Lower plots: DMP methylation type for CCC, *Adb*: Absolute value of δ β, *DMP*: Differentially methylated probe, *CCC*: Clear cell carcinoma, *EC*: Endometroid carcinoma, *HGSC*: High-grade serous carcinoma, *MC*: Mucinous carcinoma. Created in R-package "ggplot"

Figure 20 Percentage of samples for CCC showing CNA

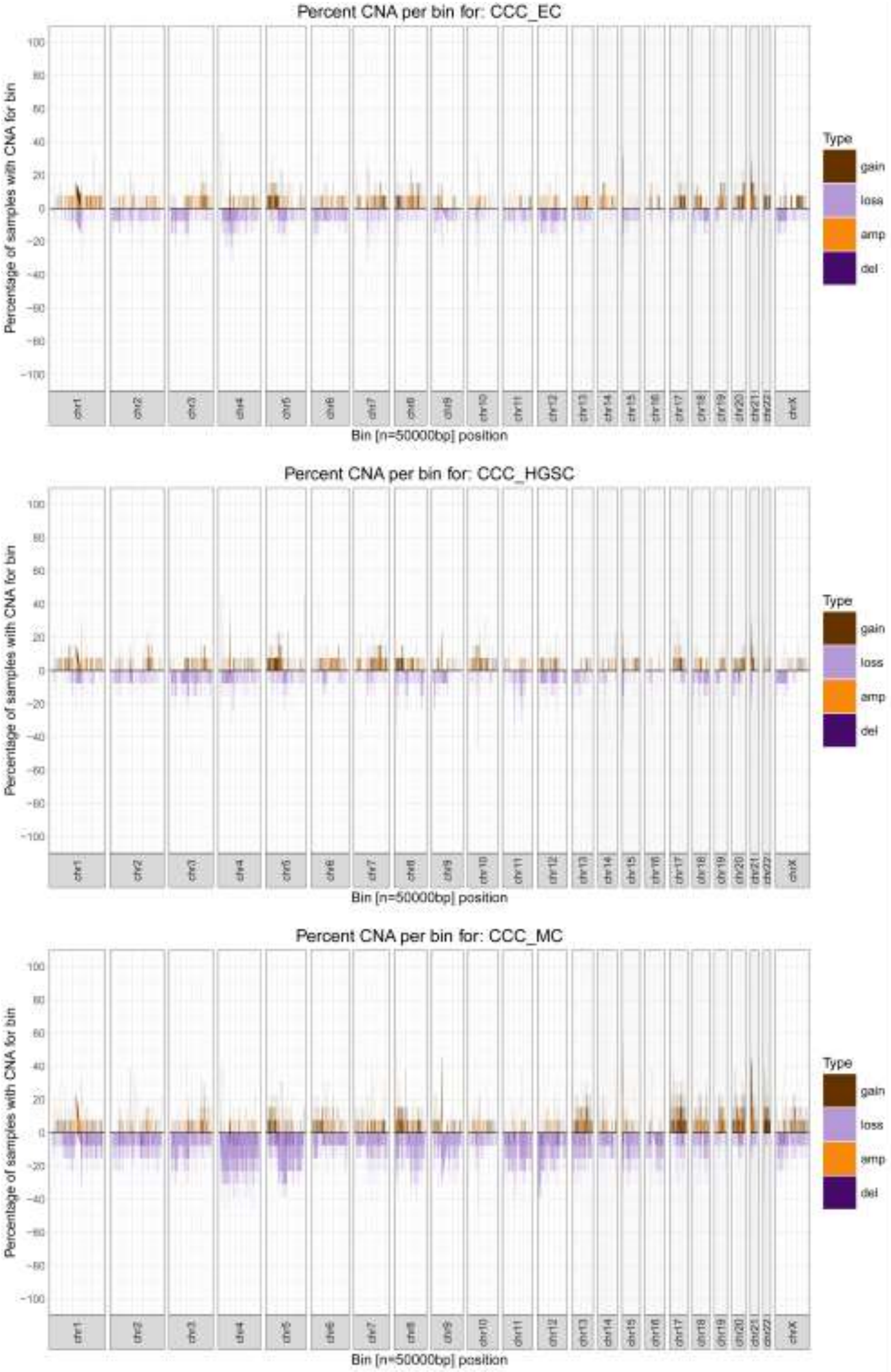

Bins: width=50000bp, *CNA:* Copy number aberrations, *Gain*: intensity>0.3, *Amp*: intensity > 0.5, *Loss*: intensity< -0.3, Del: intensity< -1, *CCC*: Clear cell carcinoma, *EC*: Endometroid carcinoma, *HGSC*: High-grade serous carcinoma, *MC*: Mucinous carcinoma Created in R-package “ggplot”

Figure 21 Percentage of samples for EC showing CNA

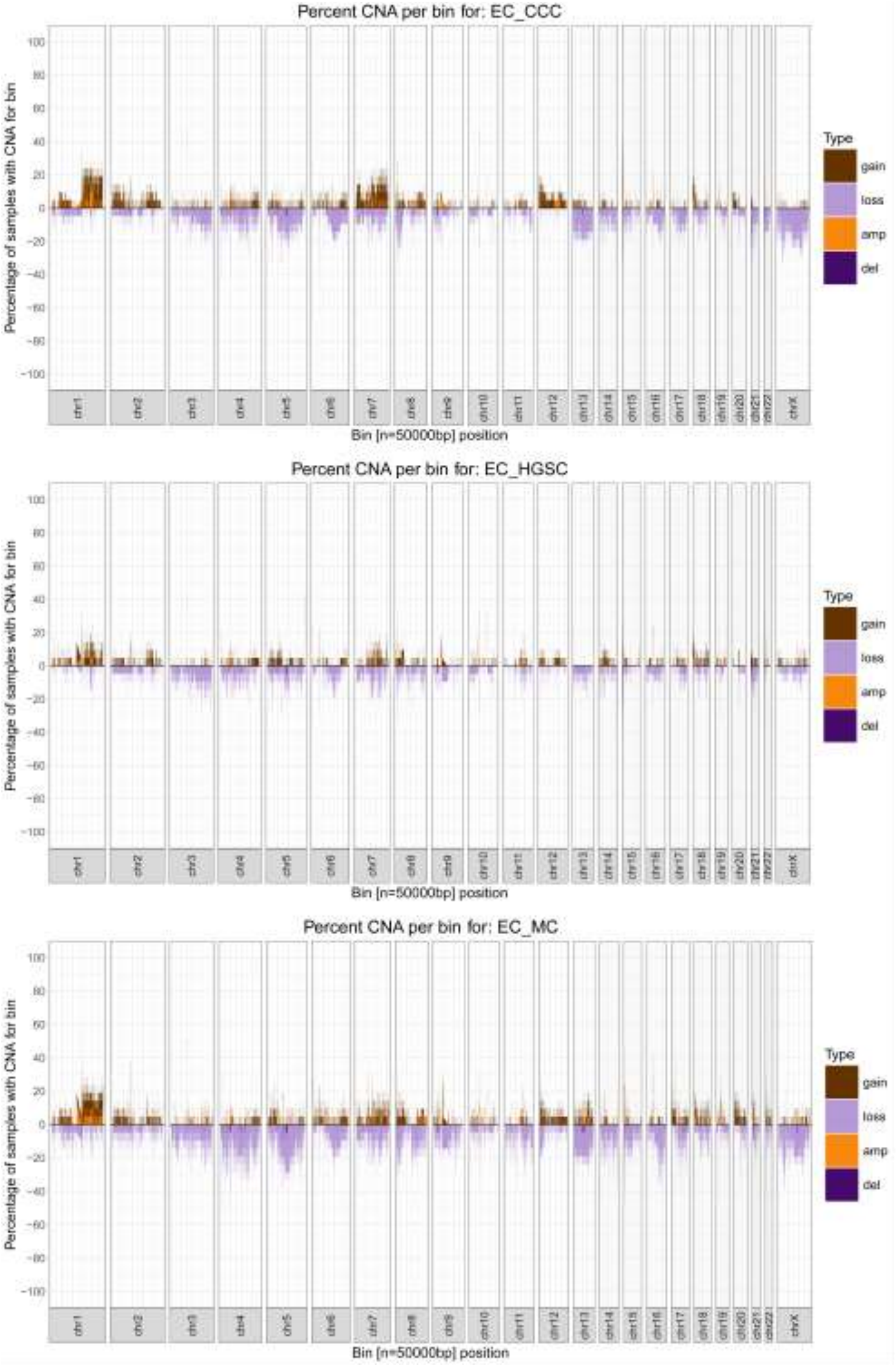

Bins: width=50000bp*, CNA:* Copy number aberrations, *Gain*: intensity>0.3, *Amp*: intensity > 0.5, *Loss*: intensity< -0.3, Del: intensity< -1, *CCC*: Clear cell carcinoma, *EC*: Endometroid carcinoma, *HGSC*: High-grade serous carcinoma, *MC*: Mucinous carcinoma Created in R-package "ggplot"

Figure 22 Percentage of samples for HGSC showing CNA

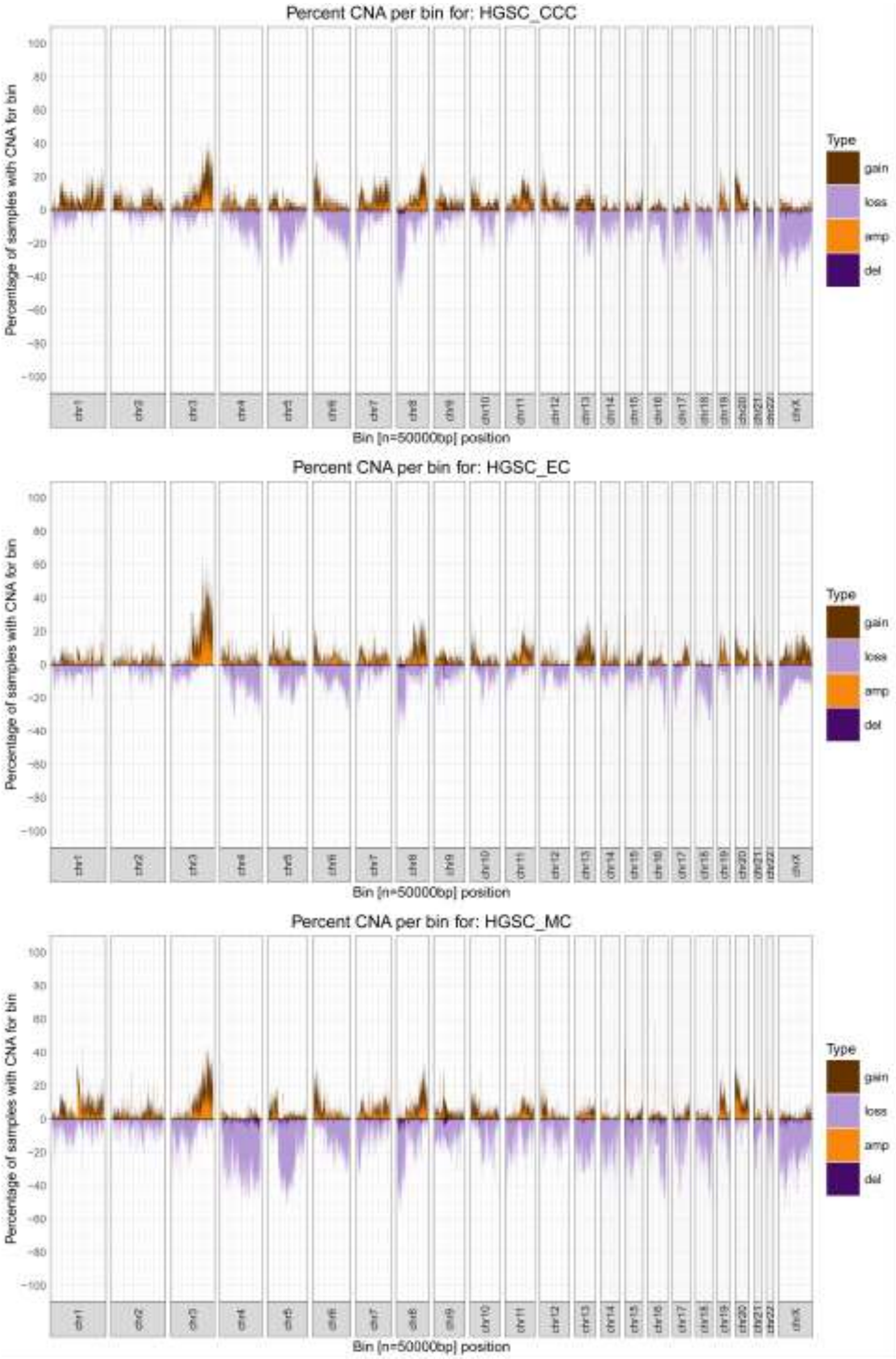

Bins: width=50000bp, *CNA:* Copy number aberrations, *Gain*: intensity>0.3, *Amp*: intensity > 0.5, *Loss*: intensity< -0.3, Del: intensity< -1, *CCC*: Clear cell carcinoma, *EC*: Endometroid carcinoma, *HGSC*: High-grade serous carcinoma, *MC*: Mucinous carcinoma Created in R-package "ggplot"

Figure 23 Percentage of samples for MC showing CNA

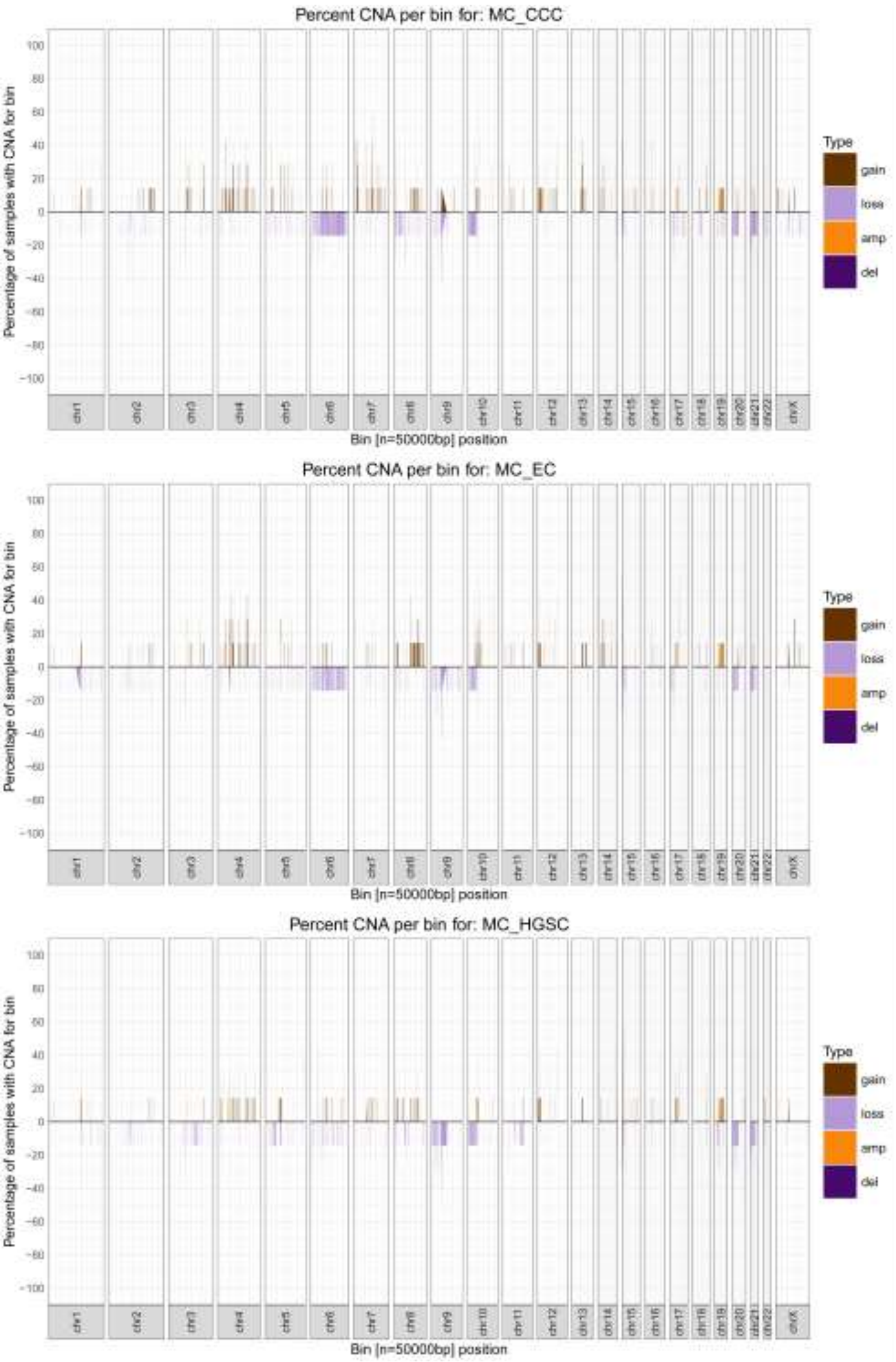

Bins: width=50000bp*, CNA:* Copy number aberrations, *Gain*: intensity>0.3, *Amp*: intensity > 0.5, *Loss*: intensity< -0.3, Del: intensity< -1, *CCC*: Clear cell carcinoma, *EC*: Endometroid carcinoma, *HGSC*: High-grade serous carcinoma, *MC*: Mucinous carcinoma Created in R-package "ggplot"

Table 18 The number of significant GISTIC lesions and regions in the training cohort

Contrast	Significant lesions	Gain	Loss	Significant regions	Gain	Loss
CCC – EC	0	0	0	0	0	0
CCC – HGSC	2	2	0	0	0	0
CCC – MC	3	1	2	38	38	0
EC – CCC	3	2	1	15	15	0
EC – HGSC	4	3	1	3	3	0
EC – MC	11	8	3	20	19	1
HGSC – CCC	30	23	7	180	44	126
HGSC – EC	34	16	18	125	64	61
HGSC – MC	41	32	9	273	71	202
MC – CCC	2	2	0	10	2	8
MC – EC	2	2	0	60	2	58
MC – HGSC	4	4	0	6	4	2

Significant lesions: q.value < 0.05, *Significant regions*: frequency > 25%, fdr < 0.05, *CCC*: Clear cell carcinoma, *EC*: Endometroid carcinoma, *HGSC*: High-grade serous carcinoma, *MC*: Mucinous carcinoma

Table 19 Differentially expressed genes present in GISTIC CNA region

**Contrast**	**DEGs**	**Gistic region genes**	**DEGs within gistic regions**	**Gain – Upregulated**	**Loss – Downregulated**	**Gain-Downregulated, Loss - Upregulated**
CCC – EC	0	0	0	0	0	0
CCC – HGSC	1309	27	1	0	1	0
CCC – MC	826	39	2	0	0	2
EC – CCC	733	29	0	0	0	0
EC – HGSC	119	68	0	0	0	0
EC – MC	162	311	0	0	0	0
HGSC – CCC	1314	3216	104	4	61	39
HGSC – EC	130	3882	9	2	6	1
HGSC – MC	772	3253	60	1	41	18
MC – CCC	797	29	0	0	0	0
MC – EC	148	17	0	0	0	0
MC – HGSC	745	1041	14	0	5	4

DEG: Differentially expressed gene, *Upregulated*: log2FC > 1, Benjamini-Hochberg <0.05, *Downregulated*: log2FC < 1, Benjamini-Hochberg <0.05, *CCC*: Clear cell carcinoma, *EC*: Endometroid carcinoma, *HGSC*: High-grade serous carcinoma, *MC*: Mucinous carcinoma

Figure 24 GISTIC CNA analysis results for CCC

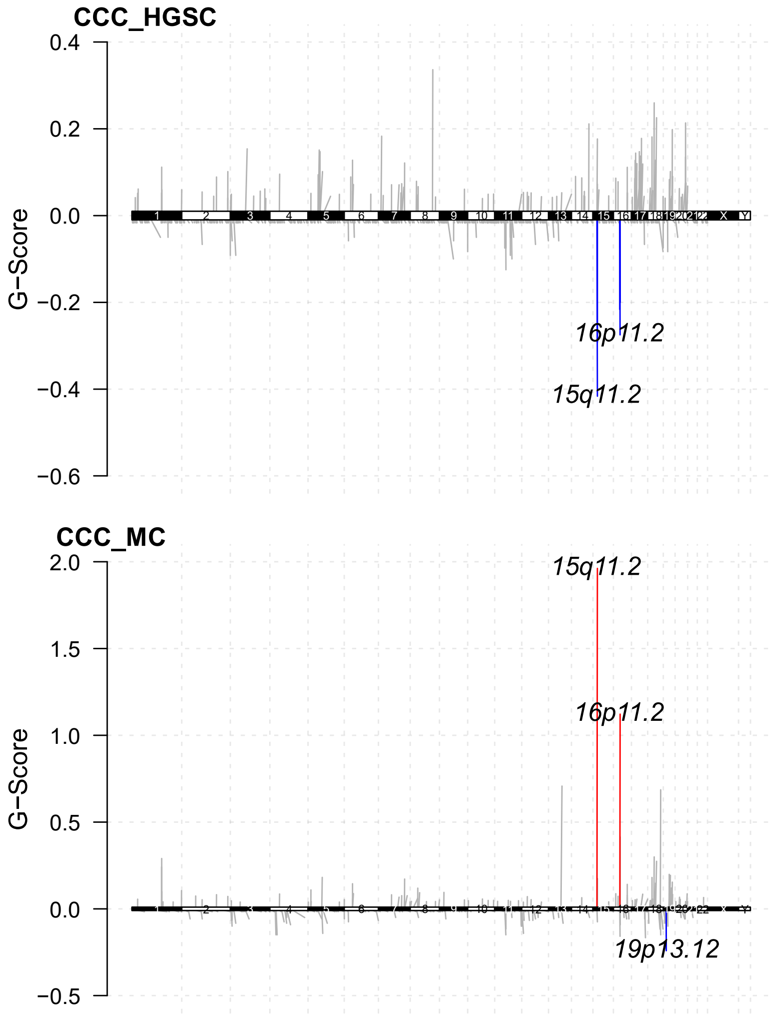

Red peaks represent CNA gains, blue peaks represent CNA losses. Cytoband name for plotted peak at end of peak. No losses or gains detected for the CCC – EC comparison. *G-score*: Score based on the aberration amplitude in relation to frequency of its occurrence across samples, used by GISTIC. *CCC*: Clear cell carcinoma, *EC*: Endometroid carcinoma, *HGSC*: High-grade serous carcinoma, *MC*: Mucinous carcinoma. Created in R-package "maftools"

Figure 25 GISTIC CNA analysis results for EC

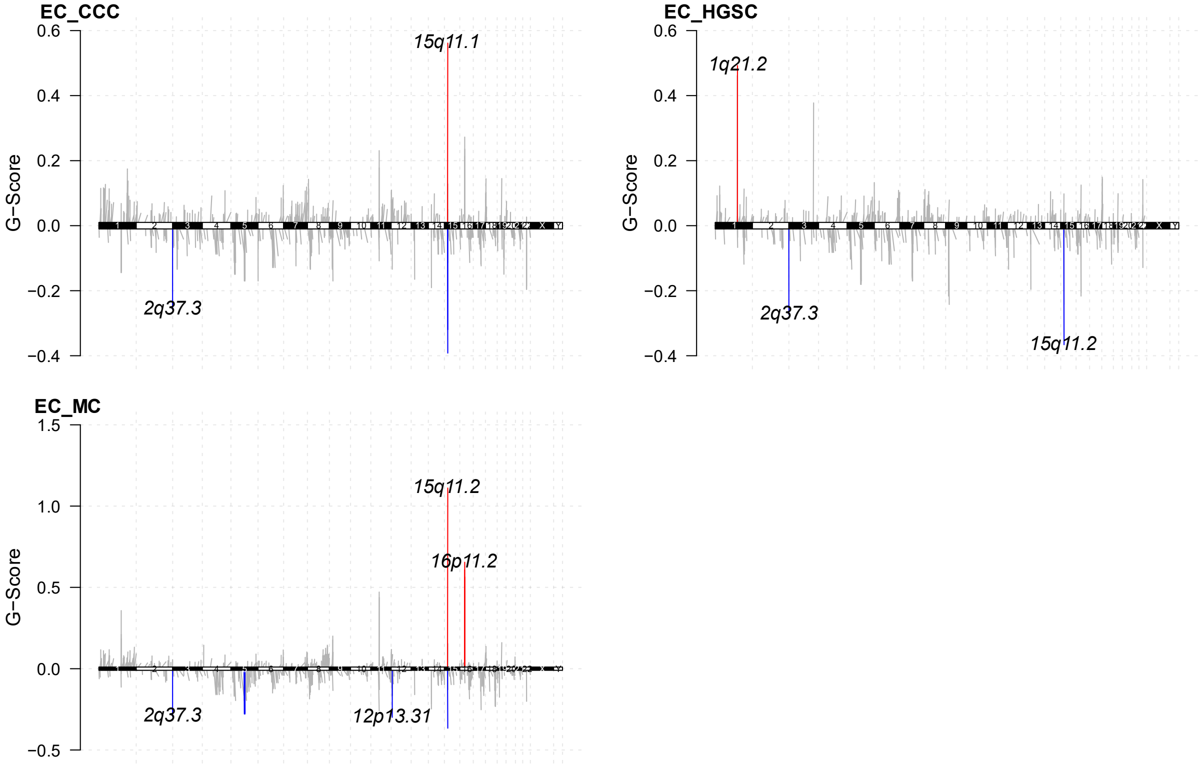

Red peaks represent CNA gains, blue peaks represent CNA losses. Cytoband name for plotted *peak* at end of peak. G-score: Score based on the aberration amplitude in relation to frequency of its occurrence across samples, used by GISTIC. *CCC*: Clear cell carcinoma, *EC*: Endometroid carcinoma, *HGSC*: High-grade serous carcinoma, *MC*: Mucinous carcinoma. Created in R-package "maftools"

Figure 26 GISTIC CNA analysis results for HGSC

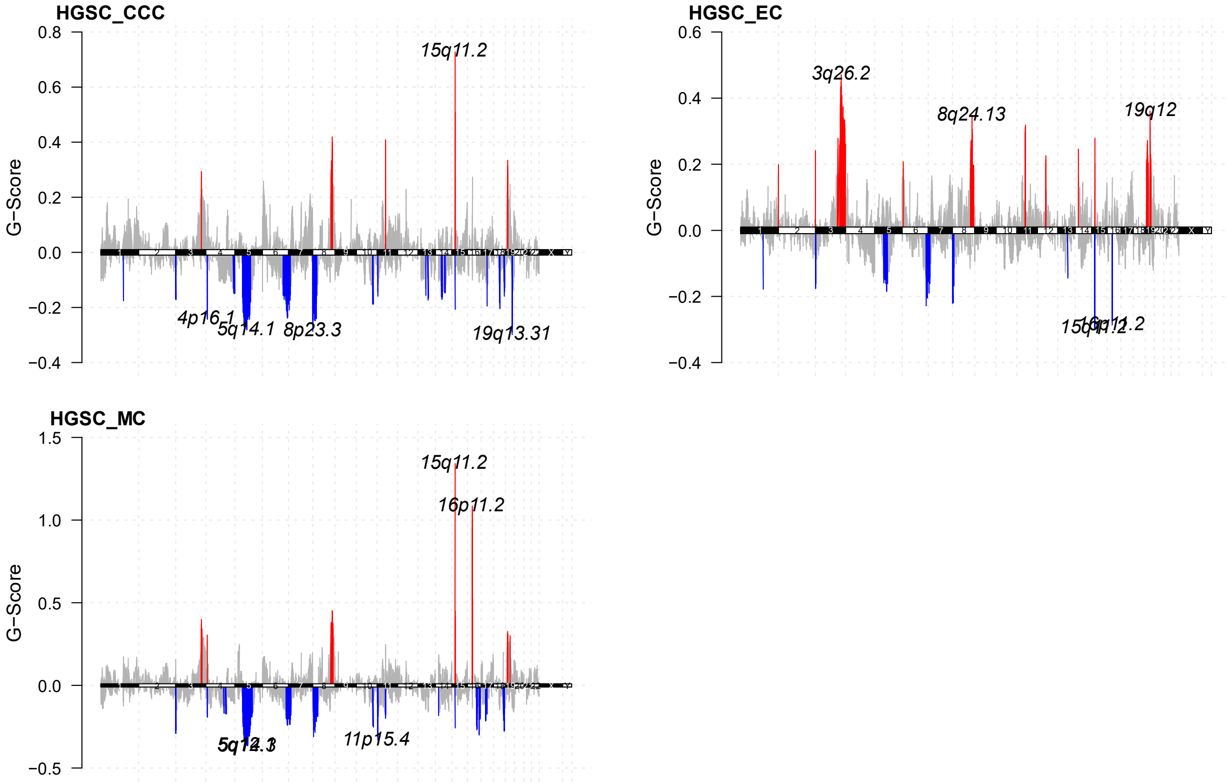

Red peaks represent CNA gains, blue peaks represent CNA losses. Cytoband name for plotted peak at end of peak. *G-score*: Score based on the aberration amplitude in relation to frequency of its occurrence across samples, used by GISTIC. *CCC*: Clear cell carcinoma, *EC*: Endometroid carcinoma, *HGSC*: High-grade serous carcinoma, *MC*: Mucinous carcinoma. Created in R-package “maftools”

Figure 27 GISTIC CNA analysis results for MC

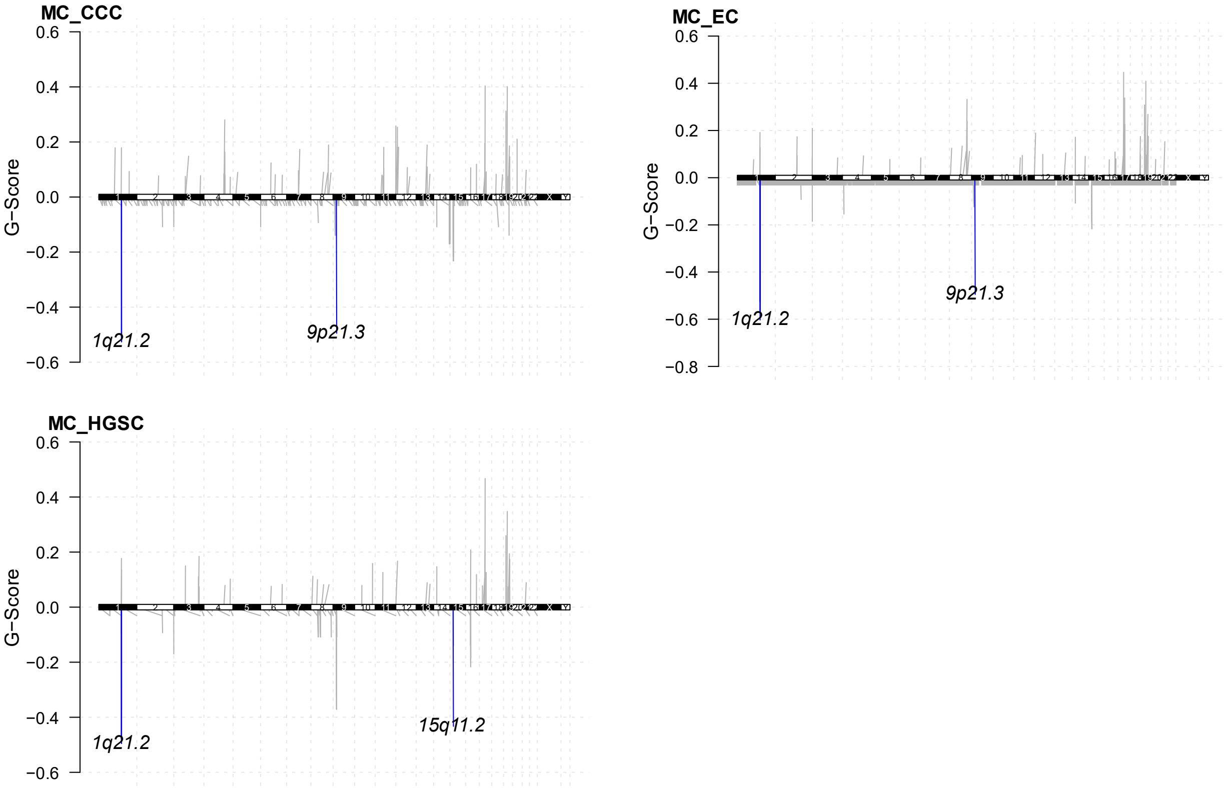

Red peaks represent CNA gains, blue peaks represent CNA losses. Cytoband name for plotted peak at end of peak. *G-score*: Score based on the aberration amplitude in relation to frequency of its occurrence across samples, used by GISTIC. *CCC*: Clear cell carcinoma, *EC*: Endometroid carcinoma, *HGSC*: High-grade serous carcinoma, *MC*: Mucinous carcinoma. Created in R-package "maftools"

Table 20 The number of HSGs found to overlap with a differentially methylated probe (DMPs) with respect to genomic position ((gene + promoter)/CpG-site)

**Comparison group**	**DMP-HSG body overlaps (% DMP)**	**DMP - HSG body hyper**	**DMP - HSG body hypo**	**DMP - HSG promoter overlaps (% DMP)**	**DMP - HSG promo hyper**	**DMP - HSG promo hypo**	**HSG body - DMP overlaps (% HSG)**	**HSG Promoter - DMP overlaps (% HSG)**
EC – CCC	18 (0.4)	10	8	7 (0.2)	6	1	18 (19.8)	6 (6.6)
HGSC – CCC	131 (0.2)	60	71	20 (0.1)	15	5	46 (45.1)	14 (13.7)
MC – CCC	61 (0.3)	11	50	12 (0.1)	7	5	32 (36.4)	7 (8.0)
CCC – EC	0 (0)	0	0	0 (0)	0	0	0 (0)	0 (0)
HGSC – EC	0 (0)	0	0	0 (0)	0	0	0 (0)	0 (0)
MC – EC	0 (0)	0	0	0 (0)	0	0	0 (0)	0 (0)
CCC – HGSC	2 (0.01)	0	2	2 (0.01)	1	1	3 (37.5)	2 (25.0)
EC – HGSC	3 (0.04)	0	3	2 (0.03)	0	2	2 (25.0)	2 (25.0)
MC – HGSC	9 (0.02)	1	8	2 (0.01)	1	1	6 (60.0)	2 (20.0)
CCC – MC	55 (0.3)	7	48	18 (0.1)	0	18	31 (44.3)	11 (15.7)
EC – MC	3 (0.0)	1	2	0 (0)	0	0	2 (4.3)	0 (0)
HGSC – MC	74 (0.2)	13	61	27 (0.1)	5	22	37 (52.1)	17 (23.9)

HSG: Histotype specific gene (classified as significantly differentially expressed gene in 3/3 possible histotype comparisons for a histotype of interest used as reference in comparisons), *DEG*: Differentially expressed gene, *DMP*: Differentially methylated probe, *DMR*: Differentially methylated region, *Hypermethylation*: $\delta\ \beta > 0.2$, *Hypomethylation*: $\delta\ \beta < -0.2$, *CCC*: Clear cell carcinoma, *EC*: Endometroid carcinoma, *HGSC*: High-grade serous carcinoma, *MC*: Mucinous carcinoma

Table 21 Genomic coordinate overlaps between differentially methylated regions and differentially expressed genes

Contrast	DMRs	DMR-DEG overlaps (%DMR)	DMR-HSG overlaps (%DMR)
CCC – EC	180	13 (7.2)	0
CCC – HGSC	560	55 (9.8)	1 (0.2)
CCC – MC	327	57 (17.4)	12 (3.7)
EC – CCC	180	13 (7.2)	1 (0.6)
EC – HGSC	108	1 (0.9)	0
EC – MC	131	11 (8.4)	10 (7.6)
HGSC – CCC	560	55 (9.8)	7 (1.3)
HGSC – EC	108	1 (0.9)	0
HGSC – MC	772	55 (7.1)	13 (1.7)
MC – CCC	327	59 (18.0)	3 (0.9)
MC – EC	131	10 (7.6)	0
MC – HGSC	772	54 (7.0)	1 (0.1)

HSG: Histotype specific gene (classified as significantly differentially expressed gene in 3/3 possible histotype comparisons for a histotype of interest used as reference in comparisons), *DMR*: Differentially methylated region, *Hypermethylation*: $\delta\ \beta > 0.2$, *Hypomethylation*: $\delta\ \beta < -0.2$, *CCC*: Clear cell carcinoma, *EC*: Endometroid carcinoma, *HGSC*: High-grade serous carcinoma, *MC*: Mucinous carcinoma

Table 22 HSGs with expression significantly correlated with gene-level CpG-site DNA-methylation.

**Name**	**Total HSGs**	**HSGs significantly correlated with promoter methylation (% HSG)**	**Significant positively correlated promoter methylation**	**Significant negatively correlated promoter methylation**	**HSGs significantly correlated with gene-body methylation (% HSG)**	**Significant positively correlated gene-body methylation**	**Significant negatively correlated gene-body methylation**
CCC	167	20 (12.0)	2	18	41 (24.6)	18	32
EC	0	0	0	0	0	0	0
HGSC	20	3 (15.0)	0	3	7 (35.0)	7	0
MC	96	14 (15.6)	1	14	35 (36.5)	21	18

Numbers indicate no. genes with CpG site methylation significantly negatively or positively correlated with gene expression (absolute Pearssons correlation coefficient > 0.5, FDR < 0.05) for either gene-promoter or gene-body region. *HSG*: Histotype specific gene (classified as significantly differentially expressed gene in 3/3 possible histotype comparisons for a histotype of interest used as reference in comparisons), *HSG*: Histotype specific gene (classified as significantly differentially expressed gene in 3/3 possible histotype comparisons for a histotype of interest used as reference in comparisons), *CCC*: Clear cell carcinoma, *EC*: Endometroid carcinoma, *HGSC*: High-grade serous carcinoma, *MC*: Mucinous carcinoma

Figure 28 Pearson correlation coefficient plot for CpG sites overlapping HSGs for CCC

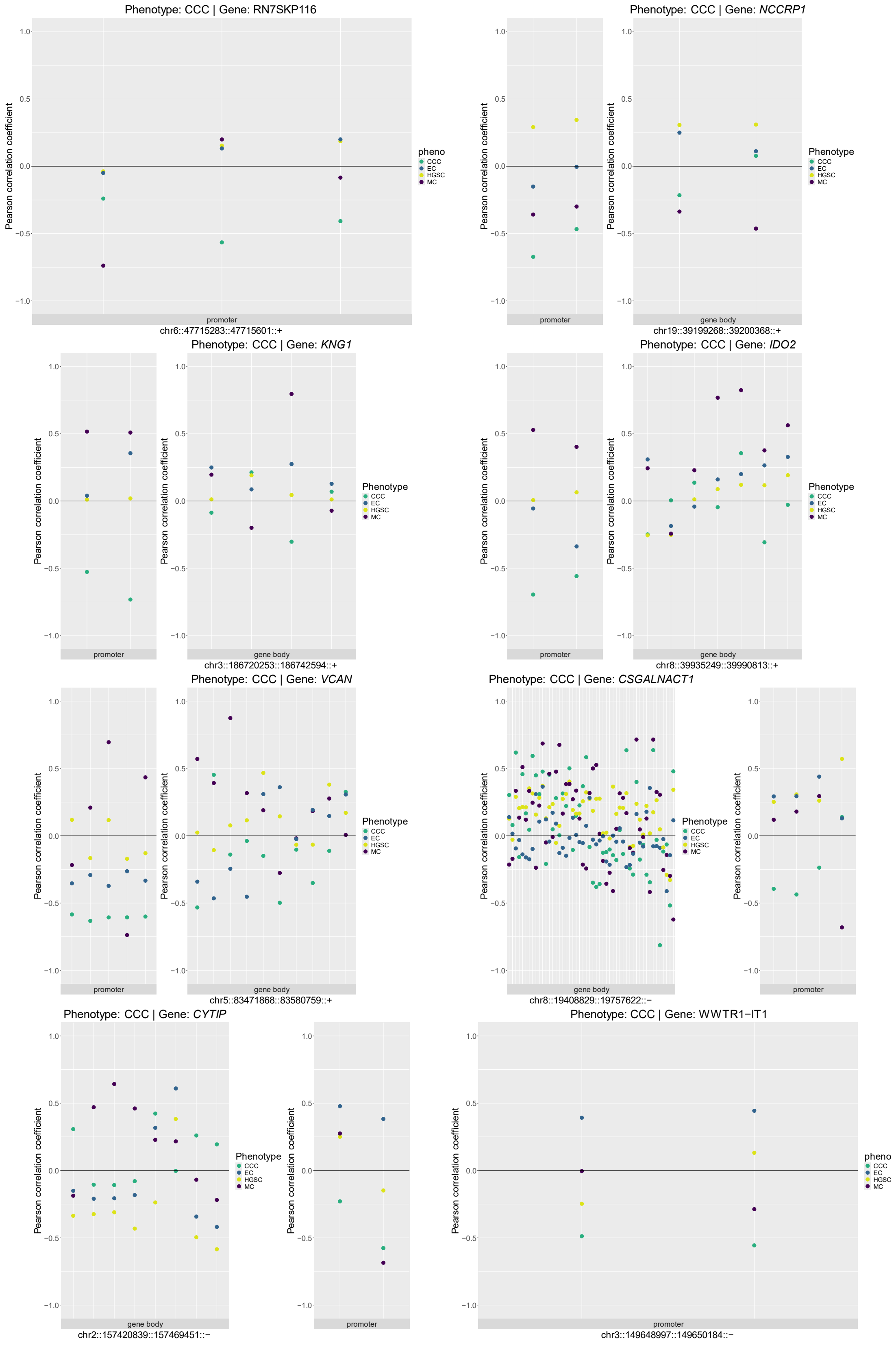

Top 8 HSGs (based on median distance between correlation coefficients for CpG sites in promoter region) for CCC. Dots show Pearson correlation coefficient for *CpG-site* DNA-methylation (*β)* in relation to gene expression (log2 normalized counts) for samples belonging to a histotype. HSGs depicted have at least one CpG site significantly negatively correlated *(spearman correlation coefficient < -0.5, adjusted p < 0.05)* with expression in the promoter region (2000bp before TSS, *50*bp after TSS). *HSG*: Histotype specific gene (classified as significantly differentially expressed gene in 3/3 possible histotype comparisons for a histotype of interest used as reference in comparisons), *CCC*: Clear cell carcinoma, *EC*: Endometroid carcinoma, *HGSC*: High-grade serous carcinoma, *MC*: Mucinous carcinoma

Figure 29 Pearson correlation coefficient plot for CpG sites overlapping HSGs for MC

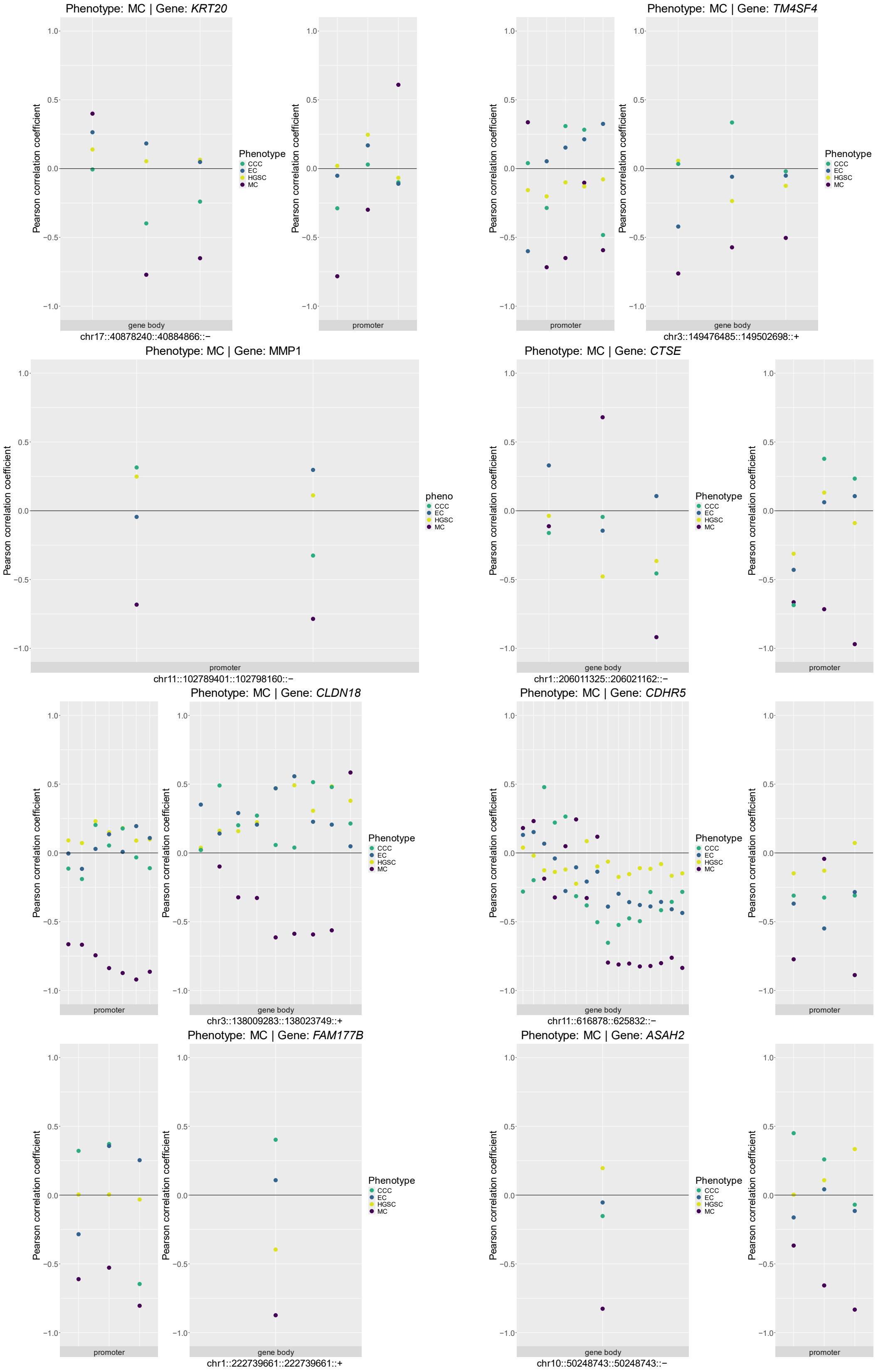

Top 8 HSGs (based on median distance between correlation coefficients for CpG sites in promoter region) for MC. Dots show Pearson correlation coefficient for *CpG-site* DNA-methylation (*β)* in relation to gene expression (log2 normalized counts) for samples belonging to a histotype. HSGs depicted have at least one CpG site significantly negatively correlated *(spearman correlation coefficient < -0.5, adjusted p < 0.05)* with expression in the promoter region (2000bp before TSS, *50*bp after TSS). *HSG*: Histotype specific gene (classified as significantly differentially expressed gene in 3/3 possible histotype comparisons for a histotype of interest used as reference in comparisons), *CCC*: Clear cell carcinoma, *EC*: Endometroid carcinoma, *HGSC*: High-grade serous carcinoma, *MC*: Mucinous carcinoma
